# Diagnostic accuracy of the Voluntary Breath-Hold Test to discriminate normal vs. abnormal spirometry: a two-gate study

**DOI:** 10.1016/j.bjane.2026.844737

**Published:** 2026-02-11

**Authors:** Gustavo Périssé Moreira Veras, Amanda Périssé Maia Veras, Thiago Prudente Bartholo, Agnaldo José Lopes, Claudia Henrique da Costa, Rogerio Rufino

**Affiliations:** a Universidade do Estado do Rio de Janeiro, Rio de Janeiro, RJ, Brazil; b Universidade Estácio de Sá, Rio de Janeiro, RJ, Brazil

**Keywords:** Breath holding, Diagnostic accuracy, Pulmonary function, Respiratory function tests, Screening, Spirometry

## Abstract

**Introduction:**

The Voluntary Breath-Hold Test (VBHT) is a simple, bedside, and rapid assessment that measures the duration of voluntary apnea. It has shown potential for clinical use, including as a triage tool prior to spirometry in primary care. However, its diagnostic accuracy in detecting abnormal pulmonary function before non-thoracic surgeries has not been established. This study aimed to determine the correlation between VBHT results, Maximal Voluntary Apnea Inspiratory Time (MVAIT) and Maximal Voluntary Apnea Expiratory Time (MVAET), and spirometry, the reference test for assessing pulmonary function.

**Methods:**

This diagnostic-accuracy study included adults with normal spirometry and those with obstructive or restrictive ventilatory defects. Participants were divided into normal and abnormal spirometry groups. Maximal Voluntary Apnea Inspiratory Time (MVAIT) and Expiratory Time (MVAET) were evaluated using Receiver Operating Characteristic (ROC) curve analysis to assess their accuracy in distinguishing between normal and abnormal spirometry patterns.

**Results:**

The study included 293 participants. MVAIT and MVAET were significantly lower for the abnormal (median: 29.32 s; 95% Confidence Interval [95% CI]: 25.99‒32.35 s and median: 20.40 s; 95% CI: 18.66‒22.88 s) than for the normal (median: 47.55 s; 95% CI: 43.93‒51.87 s and median: 28.53 s; 95% CI: 26.74‒30.63 s) group. For the prediction of normal spirometry, MVAIT ≥ 45.49 s and MVAET ≥ 32.86 s had sensitivity and specificity of (90.43%, 55.06%) and (90.43%, 33.71%), respectively.

**Conclusion:**

VBHT is a bedside, low-cost, and safe method that shows moderate-to-good discriminative ability for identifying abnormal spirometry results. As an innovative adaptation of a long-known physiological maneuver, VBHT may serve as a rapid preliminary triage (rule-out) tool prior to formal spirometry, pending external validation in preoperative populations.

**CTRI Register Number - ReBEC (Registro Brasileiro de Ensaios Clínicos):**

RBR-8hknmn.

## Introduction

Pulmonary dysfunction is a major cause of morbidity and prolonged hospital stay after non-thoracic surgery, particularly among patients with Chronic Obstructive Pulmonary Disease (COPD).^1^, ^2^, ^3^ Spirometry is the most widely used test for assessing lung function, especially through measurements such as Forced Vital Capacity (FVC). Identifying patients with abnormal spirometry before surgery is essential for optimizing perioperative management and reducing respiratory risk.^2^

Spirometry requires its own equipment, trained technicians, and interpretation by a specialist physician, which can limit its availability immediately before surgery, particularly in emergency abdominal, vascular and orthopedic surgeries.^4^ The Voluntary Breath-Hold Test (VBHT) is a simple, inexpensive, bedside, and rapid assessment that may provide useful information about ventilatory function when spirometry is not readily available.

Apnea is defined as the temporary cessation of gas exchange between the lungs and the atmosphere.^5^ In breath-holding, there is a period without respiratory sensation or electromyographic activity of the respiratory muscles after the onset of voluntary apnea. The period between the onset of apnea and the onset of the sensation of dyspnea can be considered a “comfort phase”. After a few seconds, electromyographic activity of the diaphragm and sensation of dyspnea (uncomfortable awareness of difficulty breathing) evolve and progressively increase to the endpoint of apnea (breaking point), constituting the “struggle phase”.^5^ Expiration essentially involves passive recoil, but in voluntary breath-holding, voluntary muscles may contribute to keeping the chest open against the recoil force, which is not explained only by closure of the glottis and airways.^6^

VBHT is a simple and rapid procedure that measures the duration of voluntary apnea after a maximal inspiration, using a low-cost stopwatch. The maximum duration of voluntary apnea varies among individuals and depends on chemical and nonchemical stimuli.^7^ Previous studies have evaluated VBHT in limited clinical contexts, suggesting potential usefulness^6^ in the assessment and follow-up of patients with COPD.

This study aimed to determine whether the results of the VBHT are consistent with spirometry findings in healthy individuals and in patients with ventilatory disorders, assessing its diagnostic ability for identifying abnormal pulmonary function.

## Methods

This was a two-gate (case-control) diagnostic-accuracy study conducted at a tertiary, university-affiliated hospital between January and December 2023. In the design, participants with known obstructive or restrictive ventilatory defects (cases) and healthy volunteers with normal spirometry (controls) were recruited through separate pathways, both from the same institution. Pulmonologists analyzing spirometry results were blinded to VBHT outcomes, and the technician conducting the VBHT had no access to spirometry data. The exclusion criteria were pregnancy; cognitive impairment and behavioral disorders; use of bronchodilators, stimulant and/or illicit drugs, or use of central nervous system depressant drugs within 24 h before the examination; pain or infectious symptoms that compromised the examination; hemoptysis; pneumothorax; cardiopulmonary instability; myocardial infarction or pulmonary embolism within the past six months; cerebral, thoracic, or abdominal aneurysm; eye surgery within the past two months; previous thoracic surgery; abdominal surgery within the past 2 months; or use of continuous home oxygen therapy.

The local ethics committee approved the study (CAAE 62652722.3.0000.5259), and all participants provided written informed consent. Each participant was interviewed, and data was collected using a standardized questionnaire that included demographic data, smoking history, comorbidities, use of medicines, and labor information.

The VBHT was performed by a trained investigator using a digital stopwatch AK68 (AKSO, Brazil). The maximal voluntary apnea inspiratory and expiratory times (MVAIT and MVAET, respectively) were measured. The VBHT was performed with participants seated, wearing a nose clip, and breathing through a mouthpiece to maintain tidal volume control. After two tidal cycles, participants were instructed to perform a maximal inspiration and hold their breath until intolerable discomfort (MVAIT), or a maximal expiration followed by breath-holding at residual volume (MVAET). Each participant received a brief demonstration and two practice trials before measurement. The test stopped if dizziness, coughing, or discomfort occurred. For MVAIT determination, participants inhaled from tidal volume to total lung capacity and then held their breath. For MVAET determination, participants breathed at tidal volume, inspired to total lung capacity, and exhaled to residual volume before holding their breath. Each maneuver was repeated twice within a 5-minute interval, and the best result was recorded. VBHT and spirometry were performed sequentially with a rest period between tests to avoid fatigue. Inter-operator reliability was evaluated in a pilot subset (n = 30), showing excellent agreement between technicians (ICC = 0.96 for MVAIT and 0.94 for MVAET). Spirometry was performed using a computerized spirometer (Spirom-3; Codax Medica, Brazil) according to the American Thoracic Society/European Respiratory Society (ATS/ERS) guidelines^7^ and published reference values.^8^ All spirometry results were independently analyzed by two experienced pulmonologists.

Data analysis was performed using GraphPad Prism version 10.0.2 for Windows (GraphPad Software, Boston, Massachusetts, USA). Continuous variables are presented as mean ± Standard Deviation (SD), median with interquartile range, or 95% Confidence Interval (95% CI), as appropriate. Parametric data were analyzed using analysis of variance (ANOVA), whereas non-parametric data were analyzed using the Kruskal-Wallis test and Spearman’s correlation. Categorical variables were compared using Fisher’s exact test. The sample-size calculation was based on estimating an expected Area Under the ROC Curve (AUC) of approximately 0.75 with a precision of ±0.05, using α = 0.05 and 80% power, resulting in a minimum required sample of 245 participants. The quality of the spirometry results was assessed using Cohen's kappa coefficient. Diagnostic accuracy of MVAIT and MVAET for identifying normal and abnormal spirometry was assessed using Receiver Operating Characteristic (ROC) curve analysis, with internal validation by bootstrap resampling (1,000 iterations). Statistical significance was set at p < 0.05 for all analyses. This study adheres to STARD 2015 guidelines; the completed checklist is provided in Supplementary Material 1.

## Results

**Figure 1:**
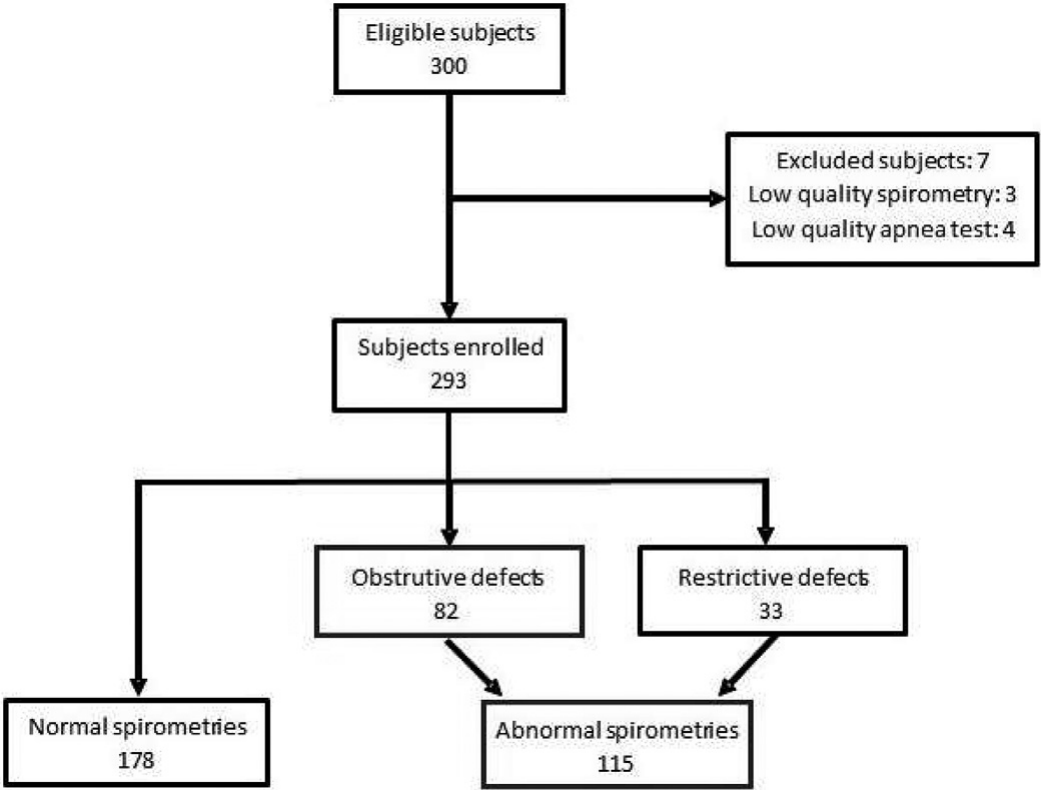
Study flowchart. A total of 300 eligible participants were screened. Seven were excluded due to low-quality spirometry (n = 3) or low-quality Voluntary Breath-Hold Tests (VBHT; n = 4). The final sample comprised 293 participants: 178 with normal spirometry and 115 with abnormal spirometry (82 obstructive and 33 restrictive ventilatory defects).

A total of three hundred individuals met the inclusion criteria and provided written informed consent (Fig. 1). Seven participants were excluded due to low-quality spirometry (not meeting ATS/ERS quality standards) or inability to perform the Voluntary Breath-Hold Test (VBHT). The final analysis included 293 participants, of whom 185 (63.1%) were female. The demographic and clinical characteristics of the study population and the corresponding VBHT results are summarized in Table 1.

**Table 1:** Data of all groups.

	**All participants** **(n = 293)**	**Normal spirometry** **(n = 178)**	**Abnormal spirometry** **(n = 115)**	**p-value**
**Sex:** Female (%)	185 (63)	105 (59)	80 (70)	0.0824
**Ethnicity:** White (%)	145 (49)	90 (51)	55 (48)	0.7198
**Age (years), median (95% CI)**	55 (49‒59)	50 (46‒51)	55 (52‒59)	0.004
**BMI (kg/m^2^), median (IQR)**	27.18 (23.78‒32.84)	27.50 (24.22‒34.10)	26.77 (22.57‒31.44)	0.033
**MVAIT (s)**				
Median (IQR)	38.06 (25.97‒54.56)	47.55 (34.32‒64.17)	29.32 (19.63‒36.94)	< 0.0001
HL diff (95% CI)			14.6 (10.4–19.5)	
**MVAET (s)**				
Median (IQR)	25.62 (19.27‒32.73)	28.53 (22.80‒36.44)	20.40 (15.72‒26.93)	< 0.0001
HL diff (95% CI)			6.6 (4.3–8.8)	

BMI, Body Mass Index; IQR, Interquartile Range; CI, Confidence Interval; MVAIT, Maximal Voluntary Apnea Inspiratory Time; MVAET, Maximal Voluntary Apnea Expiratory Time; HL diff, Hodges-Lehmann median difference (estimated difference in medians between normal and abnormal spirometry groups). Non-parametric variables were analyzed using the Mann-Whitney *U* test, and categorical variables using Fisher’s exact test. Participants with obstructive or restrictive ventilatory disorders were grouped under “abnormal spirometry” for analysis.

Participants were initially classified according to spirometry results into normal, obstructive ventilatory disorder, or restrictive ventilatory disorder groups, based on the consensus of two independent experts (Cohen’s kappa = 0.881; 95% CI: 0.826–0.936). For patients with obstructive disorders, the median MVAIT and MVAET were 31.61 s (Interquartile Range [IQR], 22.71–37.88 s) and 20.96 s (IQR: 15.50–28.96 s), respectively. For those with restrictive disorders, the median MVAIT and MVAET were 26.74 s (IQR: 17.44–33.71 s) and 20.10 s (IQR: 15.93–25.04 s), respectively. No statistically significant differences were observed between the obstructive and restrictive groups in MVAIT (p = 0.125) or MVAET (p = 0.479) (Table 2). Similarly, there were no significant differences in sex, ethnicity, or BMI between these two groups. Therefore, participants with abnormal spirometry results (obstructive or restrictive) were combined into a single abnormal group for subsequent analyses. To complement the non-parametric comparisons, we estimated the Hodges-Lehmann median differences between groups. For MVAIT, the median difference between normal and abnormal spirometry was 14.6 s (95% CI 10.4–19.5 s). For MVAET, the Hodges-Lehmann difference was 6.6 s (95% CI 4.3–8.8 s), further supporting the marked separation between groups.

**Table 2:** Data of the abnormal group.

Abnormal group (n. 115)	Obstructive disorder (n = 79)	Restrictive disorder (n = 36)	p-value
MVAIT (s) Median (IQR)	31.61 (22.71-37.88)	26.74 (17.44-33.71)	0.1255
MVAET (s) Median (IQR)	20.96 (15.50-28.96)	20.10 (15.93-25.04)	0.4794

MVAIT, maximal voluntary apnea inspiratory time; MVAET, maximal voluntary apnea expiratory time. IQR, interquartile range.

Observation: The Kruskal–Wallis test was used for non-parametric variables.

Participants in the abnormal spirometry group showed significantly lower MVAIT (median: 29.32 s; 95% CI: 25.99–32.35 s) than those in the normal group (median: 47.55 s; 95% CI: 43.93–51.87 s) (p < 0.0001). The MVAET was also significantly lower in the abnormal group (median: 20.40 s; 95% CI: 18.66–22.88 s) compared with the normal group (median: 28.53 s; 95% CI: 26.74–30.63 s) (p < 0.0001). These findings demonstrate the discriminative capacity of the VBHT to identify abnormal spirometry patterns (Table 1).

**Figure 2:**
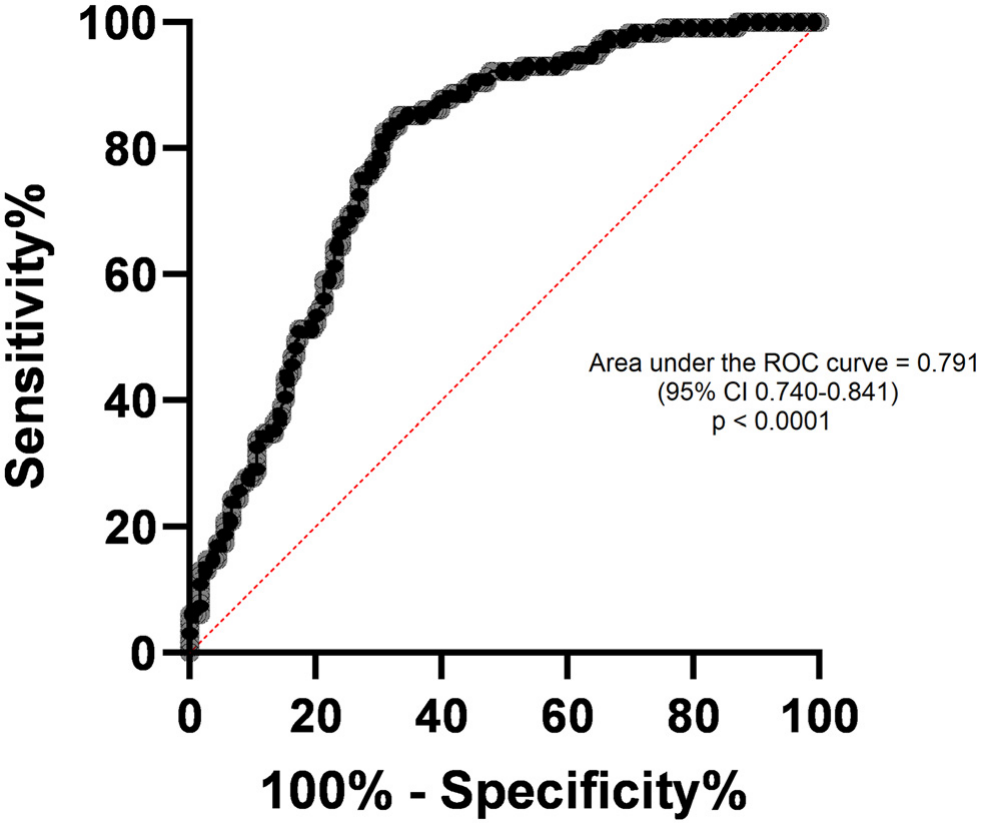
ROC curve of normal versus abnormal maximal voluntary apnea inspiratory time (MVAIT). Receiver Operating Characteristic (ROC) curve for Maximal Voluntary Apnea Inspiratory Time (MVAIT) in identifying normal spirometry results. Area Under the Curve (AUC) = 0.791 (95% CI: 0.740–0.841). The optimal cutoff (≥ 45.49 s) provided 90.43% sensitivity and 55.06% specificity. Internal validation was performed using bootstrap resampling (1,000 iterations).

**Figure 3:**
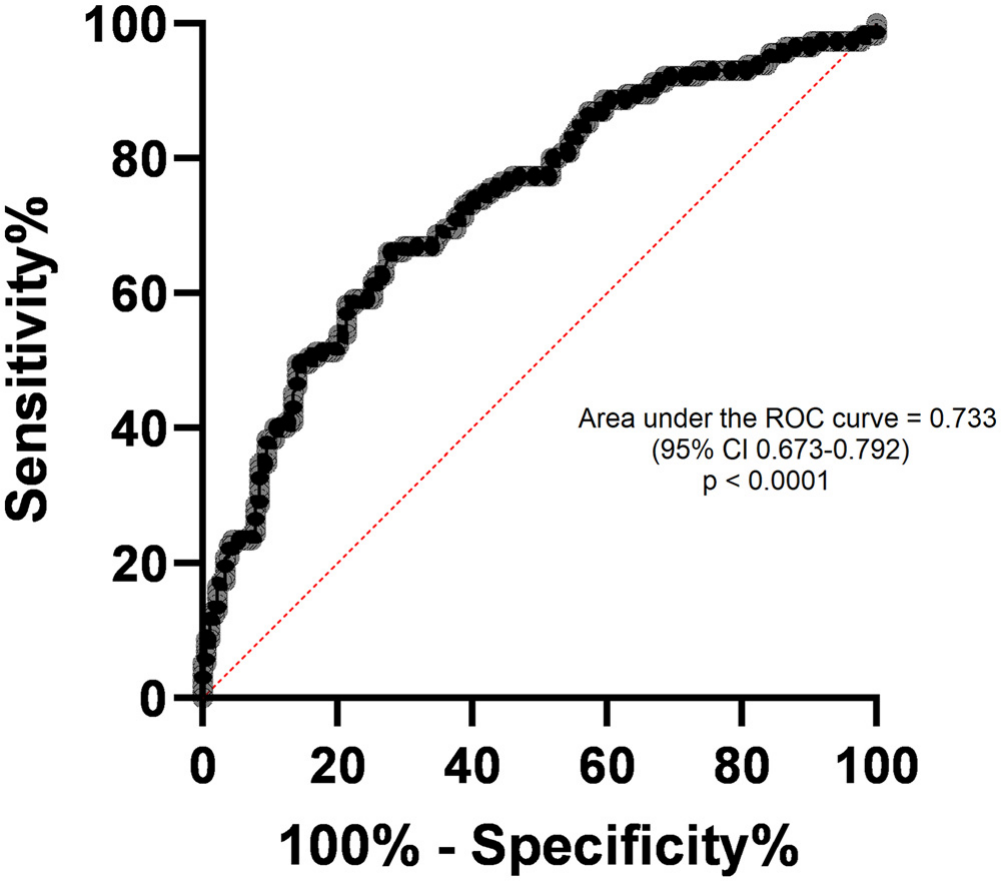
ROC curve of normal versus abnormal Maximal Voluntary Apnea Expiratory Time (MVAET). Receiver Operating Characteristic (ROC) curve for Maximal Voluntary Apnea Expiratory Time (MVAET) in identifying normal spirometry results. Area Under the Curve (AUC) = 0.733 (95% CI: 0.675–0.787). The optimal cutoff (≥ 32.86 s) provided 90.43% sensitivity and 33.71% specificity. Internal validation was performed using bootstrap resampling (1,000 iterations).

The correlations of MVAIT and MVAET with parameters of spirometry are shown in Table 3. Diagnostic accuracy was assessed by comparing the MVAIT and MVAET values between participants with normal and abnormal spirometry. The areas under the Receiver Operating Characteristic (ROC) curves were 0.791 (95% CI: 0.740–0.841) for MVAIT and 0.733 (95% CI: 0.675–0.787) for MVAET, validated by bootstrap resampling (1,000 iterations). Positive and negative predictive values (PPV and NPV) for the proposed cutoffs were 73.5% and 84.2% for MVAIT ≥ 45.49 s, and 65.9% and 80.7% for MVAET ≥ 32.86 s, respectively (Supplementary Table 1). These PPV and NPV estimates reflect the prevalence observed in this two-gate study sample and are therefore sample-dependent. For using VBHT as a diagnostic triage tool, a minimum sensitivity of 90% was established. An MVAIT ≥ 45.49 s yielded a sensitivity of 90.43% (95% CI: 83.68–94.57) and specificity of 55.06% (95% CI: 47.72–62.18). An MVAET ≥ 32.86 s showed sensitivity of 90.43% (95% CI: 83.68–94.57) and specificity of 33.71% (95% CI: 27.17–40.93) for the diagnosis of normal spirometry (Figure 2, Figure 3). Positive (LR+) and negative (LR-) likelihood ratios for MVAIT ≥ 45.49 s were 2.01 and 0.17, respectively, and for MVAET ≥ 32.86 s they were 1.36 and 0.28, respectively. The ROC-derived cutoffs for MVAIT (≥ 45.5 s) and MVAET (≥ 32.9 s) were established to maximize sensitivity for identifying normal spirometry. Internal validation was performed using bootstrap resampling (1,000 iterations). A multivariable logistic regression including age, sex, BMI, smoking status, MVAIT, and MVAET confirmed both variables as independent predictors of abnormal spirometry (p < 0.001). To further assess the diagnostic performance of VBHT, we evaluated the discrimination and calibration of the multivariable logistic regression model. The adjusted AUC was 0.74 (95% CI 0.69–0.80), obtained through bootstrap validation with 1,000 resamples, indicating preserved discriminative ability after adjustment for age, sex, BMI, smoking status, MVAIT, and MVAET. Model calibration was adequate, with a bootstrap-corrected calibration intercept of approximately 0.00 (95% CI: 0.25 to 0.26) and slope of 1.01 (95% CI: 0.73 to 1.33), demonstrating good agreement between predicted and observed probabilities. A detailed calibration curve is available in the Supplementary Material.

**Table 3:** Apnea test data and spirometry volumes and flows analysis.

	MVAIT		MVAET	
	r	p-valor	r	p-valor
CVF (L)	0.4806	< 0.0001	0.3799	< 0.0001
VEF_1_ (L)	0.3304	< 0.0001	0.1409	0.0158
VEF_1_/CVF (%)	0.0655	0.2641	-0.0228	0.6970
FEFmax (L/s)	0.3371	< 0.0001	0.1649	0.0047
VVM (L/min)	0.3253	< 0.0001	0.1352	0.0208

MVAIT, maximal voluntary apnea inspiratory time; MVAET, maximal voluntary apnea expiratory time; FVC, forced vital capacity; FEV1, forced expiratory volume in one second; FEV1/FVC, FEV1/FVC ratio; FEFmax, maximal expiratory flow rate; VVM, maximum voluntary ventilation.

Observation: Spearman's correlation was used for non-parametric variables.

There were no major complications, such as desaturation, respiratory failure, or symptoms of myocardial ischemia. The overall incidence of minor, transient adverse signs and symptoms was 20.48%. Few participants experienced coughing (6.83%), tiredness (5.12%), headache (7.85%), blurred vision (1.36%), tachycardia (3.41%), and/or transient peak hypertension (0.34%). VBHT was safe and well tolerated, with only mild, self-limited symptoms observed in 21% of participants (e.g., transient dizziness, cough, or dyspnea). Participants were observed for at least two minutes after each test, and all symptoms resolved spontaneously without any medical intervention or test interruption. The complete dataset used for all analyses is provided as Supplementary Material.

## Discussion

VBHT is a non-invasive, inexpensive, and easily reproducible bedside test with potential value as a triage (“rule-out”) tool prior to spirometry for assessing ventilatory function.^10^ During the procedure, individuals voluntarily hold their breath for as long as possible, beginning from total lung capacity (Maximal Voluntary Apnea Inspiratory Time, MVAIT) or from residual volume (Maximal Voluntary Apnea Expiratory Time, MVAET). Using a low-cost accessory device such as a stopwatch, trained staff can perform the measurements after a brief learning period. Our findings support the diagnostic ability of VBHT to discriminate normal from abnormal spirometry patterns.^7^ In a pragmatic clinical pathway, individuals with VBHT values equal to or above the proposed cut-offs may be classified as “likely normal”, whereas those with values below the cut-offs should undergo formal pulmonary function testing.

Statistical validation of VBHT by comparing the values of healthy patients with those of patients with obstructive and restrictive ventilatory disorders was necessary. The diagnosis was based on the results of spirometry and, when necessary, confirmed by whole-body plethysmography analyzed by two specialists based on pulmonary function tests.^8^ Patients with obstructive and restrictive ventilatory disorders were included into a distinct group (abnormal group), because their MVAIT and MVAET values were not significantly different.

Given the two-gate (case-control) design, the contrast between normal and abnormal participants may be artificially amplified, which can lead to overestimation of diagnostic accuracy parameters, including sensitivity and the AUC. This phenomenon, known as spectrum bias, is intrinsic to case-control diagnostic studies. Therefore, although our results support the discriminative capacity of VBHT, they require confirmation in a single-gate, prospectively recruited consecutive cohort before clinical implementation.^9^ Because participants were recruited from a pulmonary function testing environment and included healthy volunteers, the estimated diagnostic performance may not directly reflect that of a routine preoperative clinic population.

In this context, VBHT demonstrated high sensitivity but only modest specificity, a profile that aligns more closely with a triage (“rule-out”) application rather than a diagnostic replacement for spirometry. The test may help identify individuals who are very unlikely to have abnormal ventilatory patterns, potentially reducing unnecessary spirometry evaluations in selected settings. However, VBHT should not be used as a standalone diagnostic tool, and any abnormal or borderline result must still be confirmed by conventional spirometry. Because the present two-gate design may overestimate discrimination, especially sensitivity and AUC, external validation using a single-gate, consecutively recruited cohort is essential before any clinical integration.^10^ Because predictive values depend on disease prevalence, the PPV and NPV reported here are not directly transportable to routine preoperative settings, where the pretest probability of abnormal spirometry is typically lower.

The diagnostic performance of the VBHT was statistically validated by comparing values from participants with normal spirometry to those with obstructive or restrictive ventilatory disorders.^11^ Spirometry was used as the reference standard, and, when clinically indicated, whole-body plethysmography was used for diagnostic confirmation.^8^ Because MVAIT and MVAET values did not differ significantly between patients with obstructive and restrictive disorders, these participants were combined into a single abnormal group for analysis. These findings are consistent with the results reported by Barnai et al.,^12^ who demonstrated that voluntary breath-holding times correlate with ventilatory limitation in patients with cystic fibrosis, supporting the physiological rationale for VBHT as a diagnostic screening tool.

Moreover, Hedhli et al.^5^ conducted a cross-sectional study in 2021 including patients with stable COPD. Their results showed that MVAIT was significantly correlated with FEV_1_ (*r* = 0.686; p < 0.0001), FVC (*r* = 0.632) and FEV_1_/FVC ratio (*r* = 0.645). In addition, MVAIT demonstrated good discriminative power for severe forms of COPD with an area under the ROC of 0.822 (95% CI: 0.7–0.945). An MVAIT < 20.5 s allowed the detection of FEV_1_ < 50% with 96% specificity and 72% sensitivity. In the present study, lower correlation coefficients were observed, and no statistically significant correlations were found between MVAIT or MVAET and the FEV₁/FVC ratio. These differences likely reflect the broader heterogeneity of our population, which included individuals without established COPD, rather than methodological discrepancies. This correlation likely reflects the integrative nature of VBHT, which assesses chemosensitivity and voluntary respiratory control, providing complementary, not redundant, information to spirometry volumes and flows.

Dankerk et al.^2^ conducted a systematic review showing that, due to limited evidence and methodological variability, it remains unclear whether preoperative spirometry is sufficient to identify ventilatory impairment before non-thoracic surgery. The available literature remains inconclusive, with 65% of prospective studies supporting and 35% questioning the diagnostic contribution of preoperative spirometry. Nevertheless, spirometry prior to upper abdominal surgery is still recommended for individuals presenting typical symptoms of COPD according to GOLD key indicators. Our findings complement this discussion by suggesting that the VBHT may be a simple and accessible alternative for detecting abnormal spirometry patterns in the preoperative setting.

Tak et al.^3^ reported that a lower preoperative FVC was associated with the occurrence of PPCs after laparoscopic abdominal surgery, whereas FEV₁ and the FEV₁/FVC ratio were not. This finding highlights the clinical relevance of FVC as an indicator of ventilatory limitation. In the present study, MVAIT and MVAET were both significantly correlated with FVC, suggesting that the VBHT may serve as a simple diagnostic tool for identifying reduced ventilatory capacity in the preoperative evaluation of non-thoracic surgical candidates.

No major complications occurred during the performance of the VBHT, confirming that the procedure is safe and well tolerated, supporting its feasibility for use in the preoperative setting.

The present study has limitations. All participants were recruited from a single pulmonary disease monitoring center, which may limit external generalizability and introduce selection bias. The age, sex, and BMI distribution of participants was not uniform, and the sample size was not powered for subgroup analyses. The pooling of obstructive and restrictive disorders may have masked subtle differences in VBHT performance between these patterns; future studies should analyze them separately. Excluding participants with recent surgery, cognitive impairment, or medication effects likely reduced variability and may underestimate challenges in real-world settings. Additionally, spirometry was used as the reference standard rather than an independent gold standard for diagnosis. Future research should include diffusing capacity of the lungs for carbon monoxide and cardiopulmonary exercise testing to refine diagnostic characterization and further validate the VBHT as a screening tool. Incorporating Diffusion Capacity (DLCO) and cardiopulmonary exercise testing variables may help clarify how VBHT reflects overall ventilatory reserve and gas exchange efficiency, supporting the future development of composite diagnostic or preoperative screening indices.

## Conclusion

In conclusion, VBHT is a simple, reproducible, and low-cost test with potential utility as a preliminary triage tool to identify individuals unlikely to have abnormal spirometry. Its feasibility and minimal resource requirements make it appealing for preoperative screening or in settings with limited access to formal pulmonary function testing. Nevertheless, because VBHT showed high sensitivity but modest specificity and was evaluated in a two-gate design, it should not replace spirometry. Validation in a single gate, prospectively recruited population is required before VBHT can be recommended for routine clinical use.

## Abbreviations

Postoperative Pulmonary Complication (PPC); Forced Vital Capacity (FVC); Chronic Obstructive Pulmonary Disease (COPD); Voluntary Breath-Holding Test (VBHT); Maximal Voluntary Apnea Inspiratory Time (MVAIT); Maximal Voluntary Apnea Expiratory Time (MVAET); Receiver Operating Characteristic (ROC); Standard Deviation (SD); Body Mass Index (BMI); Forced Expiratory Volume in the first second (FEV_1_); Interquartile Range (IQR); Confidence Interval (CI); Perception of Dyspnea (POD); American Thoracic Society (ATS); European Respiratory Society (ERS).

## Authors' contributions

The authors contributed equally as co-first authors.

## AI assistance disclosure

No AI-assisted tools were used in the preparation, analysis, or writing of this manuscript.

## Data availability statement

The datasets generated and/or analyzed during the current study are provided as Supplementary Material.

## Declaration of competing interest

The authors declare no conflicts of interest.

## Appendix Supplementary materials

Image, application 1

## Footnotes

Supplementary material associated with this article can be found in the online version at doi:10.1016/j.bjane.2026.844737.
