# Supplementary material for: Diagnostic accuracy of the Voluntary Breath-Hold Test to discriminate normal vs. abnormal spirometry: a two-gate study
Source: Braz J Anesthesiol. 2026 Feb 11;76(3):844737. doi: 10.1016/j.bjane.2026.844737 (PMC12997292; doi:10.1016/j.bjane.2026.844737)
Supplement: Supplementary file 1 [file mmc1.pdf]

Patient	Diagnosis Spirometric	Age	Height	Weight	BMI	Gender	Self-declared Ethnicity
1	normal	82	1,63	82	31	male	white
2	normal	62	1,74	62	20	female	no-white
3	abnormal	65	1,57	59	24	male	no-white
4	abnormal	19	1,62	59	22	female	white
5	abnormal	59	1,53	85	36	female	white
6	abnormal	77	1,61	60	23	male	white
7	abnormal	26	1,63	55	21	male	white
8	abnormal	20	1,52	40	17	male	white
9	abnormal	63	1,65	80	29	female	no information
10	normal	60	1,56	98	40	female	no-white
11	abnormal	86	1,74	75	25	male	white
12	normal	59	1,53	61	26	female	white
13	normal	24	1,71	63	22	female	no-white
14	normal	53	1,61	130	50	female	no information
15	normal	38	1,58	72	29	female	no-white
16	normal	55	1,69	101	35	male	no-white
17	normal	70	1,59	66	26	female	white
18	abnormal	32	1,63	55	21	female	white
19	abnormal	74	1,57	68	28	male	white
20	normal	22	1,67	71	25	male	no-white
21	normal	71	1,69	75	26	male	white
22	abnormal	22	1,61	49	19	female	no information
23	normal	37	1,49	60	27	female	no-white
24	normal	26	1,75	74	24	male	no-white
25	normal	35	1,68	67	24	male	white
26	abnormal	80	1,59	66	26	male	no-white
27	abnormal	42	1,75	58	19	male	no-white
28	normal	69	1,51	51	22	female	white
29	abnormal	69	1,65	72	26	male	no-white
30	normal	48	1,69	83	29	female	white
31	normal	60	1,67	80	29	female	white
32	normal	38	1,81	116	35	male	white
33	abnormal	45	1,57	91	37	female	white
34	normal	29	1,51	57	25	female	no-white
35	abnormal	37	1,53	74	32	female	no-white
36	abnormal	31	1,55	42	17	female	white
37	normal	45	1,64	97	36	female	white
38	abnormal	82	1,55	60	25	female	white
39	abnormal	65	1,64	79	29	female	white
41	abnormal	78	1,74	74	24	male	white
42	abnormal	69	1,51	60	26	female	no-white
43	abnormal	52	1,75	96	31	male	no-white
44	abnormal	57	1,46	54	25	female	no information
45	normal	59	1,57	73	30	female	white
46	normal	35	1,6	61	24	female	white
47	abnormal	59	1,55	55	23	female	no-white
48	abnormal	42	1,58	59	24	female	white
49	abnormal	39	1,6	109	43	female	white
50	normal	70	1,58	67	27	female	white
51	normal	67	1,64	72	27	male	white
52	abnormal	66	1,48	74	34	female	no-white
53	normal	30	1,6	64	25	female	no-white
54	abnormal	68	1,58	62	25	female	no-white
55	abnormal	72	1,54	45	19	female	white
56	normal	60	1,62	85	32	male	no-white
57	abnormal	68	1,75	77	25	male	white
58	normal	56	1,6	73	29	female	white
59	abnormal	59	1,48	76	35	female	white

60	normal	63	1,5	76	34	female	white
61	abnormal	18	1,64	72,4	27	female	white
62	abnormal	60	1,48	70	32	female	no-white
63	normal	34	1,51	79,3	35	female	white
64	normal	49	1,75	113	37	male	no-white
65	abnormal	26	1,78	71,5	23	male	no-white
66	normal	38	1,74	93	31	male	no-white
67	normal	63	1,7	65	22	male	white
68	abnormal	68	1,74	64	21	male	no-white
69	normal	18	1,85	85	25	male	no information
70	abnormal	44	1,62	94	36	female	no-white
71	abnormal	21	1,58	58	23	male	no-white
72	abnormal	55	1,6	59	23	female	no-white
73	abnormal	47	1,7	79	27	male	white
74	normal	71	1,94	103	27	male	white
75	normal	70	1,71	105	36	male	no-white
76	abnormal	68	1,52	69	30	female	white
77	abnormal	46	1,57	67	27	female	white
78	abnormal	62	1,72	97	33	female	white
79	normal	66	1,7	57	20	male	white
80	abnormal	60	1,55	73	30	female	white
81	normal	66	1,68	67	24	male	white
82	normal	53	1,7	70	24	male	white
83	normal	39	1,77	56	18	male	no-white
84	normal	27	1,65	75	28	female	no-white
85	normal	68	1,62	75	29	male	no-white
86	normal	39	1,59	70	28	female	white
87	normal	40	1,59	90	36	female	white
88	normal	32	1,66	100	36	female	no-white
89	abnormal	58	1,52	79	34	female	no-white
90	abnormal	59	1,63	101	38	female	no-white
91	abnormal	58	1,57	80	32	female	white
92	normal	50	1,64	70,8	26	female	no-white
93	abnormal	35	1,56	88	36	female	no-white
94	normal	67	1,66	76	28	male	no-white
95	abnormal	78	1,57	78	32	female	no-white
96	abnormal	42	1,61	79	30	female	no-white
97	normal	40	1,53	88	38	female	no-white
98	normal	26	1,57	44	18	female	white
99	normal	33	1,71	91	31	female	no-white
100	normal	65	1,61	69	27	female	no-white
101	normal	58	1,69	72,5	25	male	white
102	abnormal	63	1,65	105	39	female	no-white
103	abnormal	19	1,78	48	15	male	no-white
105	normal	46	1,71	124	42	female	no-white
106	abnormal	75	1,58	57	23	female	white
107	abnormal	60	1,64	72	27	female	white
108	abnormal	66	1,62	66	25	female	white
109	abnormal	38	1,59	73	29	female	no-white
110	abnormal	78	1,75	75	24	male	white
111	abnormal	22	1,65	57	21	female	white
112	abnormal	27	1,65	47	17	female	white
113	abnormal	32	1,67	60	22	male	no-white
114	abnormal	69	1,46	52	24	female	white
115	abnormal	45	1,69	104	36	male	no-white
116	abnormal	76	1,5	50	22	female	white
117	normal	60	1,63	64	24	male	no-white
118	normal	71	1,5	77	34	female	white
119	normal	87	1,57	63	26	female	no-white

120	normal	41	1,69	76	27	female	no-white
121	normal	71	1,59	69	27	male	white
122	normal	24	1,75	64	21	male	white
123	normal	69	1,56	62	25	female	white
124	normal	37	1,64	54	20	female	white
125	abnormal	30	1,54	37	16	female	white
126	normal	29	1,72	74	25	male	white
127	normal	53	1,64	69	26	female	white
128	abnormal	21	1,53	40	17	male	no-white
129	normal	57	1,66	70	25	female	white
130	normal	46	1,7	110	38	male	no-white
131	normal	28	1,66	73	26	female	white
132	normal	57	1,68	68	24	female	white
133	normal	64	1,66	74	27	male	no-white
135	normal	37	1,67	100	36	female	no-white
136	normal	30	1,84	117	35	male	no-white
137	abnormal	57	1,6	87,2	34	female	white
138	normal	68	1,73	58	19	male	white
139	abnormal	80	1,72	80	27	male	white
140	abnormal	69	1,5	30	13	female	preta
141	normal	59	1,48	45	21	female	white
142	abnormal	52	1,5	59	26	female	no-white
143	abnormal	31	1,79	100	31	male	no-white
144	normal	52	1,62	124	47	female	no-white
145	abnormal	42	1,65	142	52	female	no-white
146	normal	53	1,6	53	21	female	no-white
147	abnormal	71	1,58	66	26	female	no-white
148	abnormal	62	1,7	92	32	male	no-white
149	normal	51	1,72	102	34	male	white
150	normal	45	1,71	130	44	female	no-white
151	abnormal	21	1,58	42	17	female	white
152	normal	37	1,7	92	32	female	no-white
153	normal	51	1,68	97	34	male	white
154	normal	45	1,7	68	24	male	no-white
155	abnormal	72	1,45	44	21	female	white
156	normal	73	1,56	90	37	female	no-white
157	abnormal	60	1,54	72	30	female	white
158	normal	71	1,78	80	25	male	no-white
159	normal	61	1,5	97	43	female	white
160	normal	69	1,71	90	31	male	no-white
161	abnormal	71	1,58	71	28	male	no-white
162	normal	54	1,8	140	43	female	white
163	normal	63	1,55	53	22	female	white
164	abnormal	71	1,55	79	33	female	no-white
165	normal	68	1,72	79	27	male	white
166	normal	72	1,56	55	23	female	white
167	abnormal	66	1,71	66	23	female	no-white
168	normal	24	1,8	62	19	male	no-white
169	normal	50	1,68	75	27	female	no-white
170	normal	65	1,81	109	33	male	no-white
171	normal	62	1,57	86	35	female	no-white
172	normal	68	1,67	95	34	male	white
173	normal	27	1,82	163	49	male	white
174	normal	67	1,56	85	35	female	white
175	normal	67	1,81	67	20	male	white
176	normal	33	1,68	64	23	female	white
177	normal	38	1,73	96	32	male	white
178	normal	31	1,61	79	30	male	white
179	normal	75	1,62	67	26	male	white

180	normal	35	1,55	72	30	female	white
181	normal	47	1,85	137	40	male	no-white
182	normal	37	1,62	80	30	female	no-white
183	normal	68	1,58	58	23	female	no-white
185	normal	27	1,66	62	22	female	white
186	normal	49	1,71	96	33	female	no-white
187	normal	22	1,7	100	35	female	no-white
188	abnormal	66	1,56	70	29	female	no-white
190	abnormal	63	1,56	65	27	female	white
191	abnormal	55	1,56	83	34	female	no-white
193	abnormal	63	1,7	80	28	female	white
194	normal	23	1,59	53	21	male	white
195	normal	40	1,78	122	39	male	no-white
196	abnormal	69	1,68	69	24	female	no-white
197	abnormal	37	1,72	80	27	male	white
198	abnormal	63	1,7	84	29	female	no-white
199	normal	60	1,74	79	26	male	white
200	abnormal	74	1,62	73	28	female	white
201	normal	34	1,71	108	37	female	no-white
202	normal	69	1,64	73	27	male	no-white
203	normal	68	1,52	57	25	female	white
204	normal	53	1,54	87	37	female	white
205	normal	66	1,67	89	32	male	white
206	normal	47	1,67	75	27	male	white
207	normal	52	1,6	82	32	female	no-white
208	normal	66	1,62	66	25	female	no-white
209	normal	30	1,42	74	37	female	no-white
210	abnormal	58	1,75	95	31	male	no-white
211	normal	23	1,51	51	22	female	white
212	normal	61	1,53	77	33	female	white
213	normal	53	1,67	101	36	female	no-white
214	normal	34	1,7	70	24	male	white
215	normal	54	1,63	101	38	female	white
216	abnormal	74	1,7	84	29	male	white
217	abnormal	78	1,65	50	18	male	white
218	normal	62	1,66	90	33	male	white
219	normal	25	1,74	114	38	female	no-white
220	abnormal	25	1,83	110	33	male	no-white
221	abnormal	67	1,59	56	22	female	white
222	abnormal	71	1,62	66	25	female	no-white
223	abnormal	59	1,68	105	37	female	no-white
224	abnormal	58	1,66	140	51	female	no-white
225	normal	23	1,6	67	26	female	white
226	normal	36	1,71	156	53	male	no-white
227	normal	22	1,59	54	21	female	no-white
228	normal	41	1,71	66	23	female	white
229	normal	68	1,54	76	32	female	no-white
230	normal	85	1,62	74	28	male	no-white
231	normal	55	1,58	64	26	female	white
232	normal	40	1,53	75	32	female	white
233	normal	69	1,57	49	20	female	white
234	normal	60	1,55	67	28	female	no-white
235	abnormal	81	1,66	55	20	male	no-white
236	normal	28	1,57	47	19	female	white
237	normal	27	1,78	130	41	female	white
238	normal	43	1,69	64	22	female	no-white
239	normal	29	1,68	60	21	female	white
240	normal	44	1,65	68	25	female	white
241	normal	35	1,55	89	37	female	white

242	normal	44	1,58	73	29	female	white
243	normal	26	1,73	77	26	male	no-white
244	normal	27	1,83	110	33	male	no-white
245	normal	29	1,78	89	28	male	white
246	normal	56	1,56	79	32	female	no-white
248	normal	30	1,6	61	24	female	white
249	normal	26	1,55	52	22	female	white
250	normal	39	1,62	60	23	female	no-white
251	normal	25	1,63	55	21	female	white
252	abnormal	24	1,58	98	39	female	no-white
253	abnormal	57	1,68	49	17	male	white
254	abnormal	79	1,58	49	20	male	no-white
255	abnormal	75	1,77	71	23	male	white
256	abnormal	62	1,64	80	30	female	no-white
257	abnormal	86	1,59	59	23	female	white
258	normal	43	1,71	107	37	female	white
259	normal	27	1,68	68	24	female	white
260	normal	59	1,87	96	27	male	no-white
261	normal	61	1,74	54	18	male	white
262	normal	33	1,63	116	44	female	no-white
263	normal	58	1,69	70	25	female	no-white
264	normal	59	1,63	94	35	male	no-white
265	normal	57	1,58	76	30	female	no-white
266	normal	49	1,77	104	33	male	no-white
267	normal	46	1,76	97	31	male	no-white
268	normal	74	1,56	54	22	female	no-white
269	abnormal	51	1,72	93	31	female	no-white
270	normal	44	1,59	93	37	female	white
271	abnormal	50	1,66	76	28	female	no-white
272	abnormal	55	1,59	40	16	female	no-white
273	abnormal	52	1,5	67	30	female	white
274	abnormal	61	1,48	54	25	female	white
275	abnormal	57	1,57	73	30	female	white
276	normal	50	1,65	70	26	female	white
277	abnormal	62	1,53	51	22	female	no-white
278	normal	52	1,74	80	26	male	no-white
279	normal	28	1,67	79	28	male	white
280	normal	63	1,76	84	27	male	no-white
281	normal	50	1,75	86	28	male	white
282	abnormal	44	1,55	90	37	female	no-white
283	normal	48	1,7	87	30	male	no-white
284	normal	44	1,65	80	29	female	white
285	normal	57	1,61	57	22	male	no-white
286	normal	65	1,53	62	26	female	white
287	normal	40	1,77	106	34	male	no-white
288	normal	61	1,63	93	35	male	no-white
289	normal	49	1,56	64	26	female	no-white
290	normal	62	1,64	95	35	female	no-white
291	abnormal	58	1,59	77	30	female	no-white
292	abnormal	57	1,7	89	31	female	white
293	abnormal	70	1,55	92	38	female	no-white
294	abnormal	66	1,66	68	25	male	no-white
295	abnormal	65	1,48	39	18	female	white
296	normal	47	1,63	78	29	female	no-white
297	normal	58	1,57	74	30	female	no-white
298	abnormal	61	1,61	147	57	female	white
299	normal	46	1,7	79	27	male	no-white
301	normal	25	1,82	80	24	male	no-white

Smoking	MVAIT1	MVAIT2	MVAET1	MVAET2	MVAIT Max	MVAET Max	FVC Pred
former	41	14.54	8.75	20.09	41	20.09	2.53
former	46.56	61.44	42.78	42.65	61.44	42.78	3.48
former	15.22	18.59	17.88	17.31	18.59	17.88	2.53
no	34.84	43.46	27.62	25.63	43.46	27.62	3.68
smoker	18.87	35.59	21.59	19.12	35.59	21.59	2.73
smoker	13.94	12.78	12.75	12.44	13.94	12.75	2.51
no	30.8	30.06	10.75	11.69	30.8	11.69	4.2
no	26.65	30.5	15.22	15.82	30.5	15.82	3.45
former	11.96	20.72	18.66	13.47	20.72	18.66	3.07
no	20	14.32	18.54	16.03	20	18.54	2.72
former	23.38	25.94	34.78	45.94	25.94	45.94	3.34
no	41.6	53.32	35.61	49.75	53.32	49.75	2.6
no	10.72	11.47	12.87	14.06	11.47	14.06	3.99
no	20.81	25.43	20.69	19.72	25.43	20.69	3.06
no	30.72	34.84	27.72	29.15	34.84	29.15	3.18
former	8.72	24.85	14.09	20.79	24.85	20.79	3.84
former	37.56	29.19	24.48	29.91	37.56	29.91	2.72
no	20.9	33.06	32.97	25.75	33.06	32.97	3.5
former	20.31	27.53	13.91	15.94	27.53	15.94	2.26
no	15.44	43.45	24.22	37.34	43.45	37.34	4.66
former	36.75	47.5	29.93	41.65	47.5	41.65	3.37
no	22.34	24.15	16.57	19.2	24.15	19.2	3.45
no	14.75	17.66	13.06	7.85	17.66	13.06	2.8
no	58.82	74.5	24.47	24.46	74.5	24.47	5.21
no	46.9	54.81	13.08	18.41	54.81	18.41	4.35
former	24.95	56.22	58.47	41	56.22	58.47	2.28
no	74.53	76.97	36.22	42.19	76.97	42.19	4.74
no	13.78	10.32	15.68	12.78	13.78	15.68	2.25
former	17.44	13.91	23.13	11.59	17.44	23.13	3.09
no	41.06	20.05	25.06	26.15	41.06	26.15	3.5
no	73.38	90.31	34.29	33.15	90.31	34.29	3.21
smoker	52.38	61.22	27.47	22.54	61.22	27.47	5.36
no	2.32	5.19	5.28	5.44	5.19	5.44	3.02
no	13.78	13.81	15.22	13.09	13.81	15.22	2.93
no	7.03	9.47	16.62	10.35	9.47	16.62	2.97
no	26.1	37.38	17.63	17.19	37.38	17.63	3.16
no	40.88	49.63	15.86	21	49.63	21	3.33
no	27.28	18.09	15.35	15.91	27.28	15.91	2.2
no	17.63	27.38	21.5	25.75	27.38	25.75	2.99
no	23	14.56	20.6	17.65	23	20.6	3.58
no	25.28	11.92	10.82	14.15	25.28	14.15	2.34
no	5.53	5.59	5.63	7.63	5.59	7.63	4.44
no	12.15	11.69	10.32	86.95	12.15	86.95	2.41
no	11.88	5.97	18.38	11.22	11.88	18.38	2.78
no	18.35	34.18	29.46	23.81	34.18	29.46	3.32
no	21.79	18.12	20.96	17.78	21.79	20.96	2.69
no	9.53	11.35	7.03	13.49	11.35	13.49	3.11
former	28.62	14.32	10.72	12.87	28.62	12.87	3.25
no	27.06	49.75	28.69	29.03	49.75	29.03	2.68
no	78.29	83.25	56.52	53.56	83.25	56.52	3.06
smoker	16.99	22.93	18.81	23.47	22.93	23.47	2.26
no	41.03	55.59	17.63	23.87	55.59	23.87	3.4
former	17.31	31.07	30.09	24.53	31.07	30.09	2.67
no	29.69	22.23	7.41	12	29.69	12	2.5
former	78.44	84.53	41.12	44.19	84.53	44.19	3.1
former	33.25	34.41	19.99	17.99	34.41	19.99	3.96
former	52.59	56.57	24.38	26.1	56.57	26.1	2.96
former	23.43	18.47	19.03	29.41	23.43	29.41	2.38

former	112.28	7.96	25.46	13.32	112.28	25.46	2.38
no	16.89	14.43	10.19	16.37	16.89	16.37	3.78
smoker	23.72	31.84	27.25	29.35	31.84	29.35	2.36
no	28.47	41.97	18.1	23.47	41.97	23.47	2.94
no	31.36	51.87	23.31	48.84	51.87	48.84	3.75
no	30.72	9.72	22.75	32.22	30.72	32.22	5.47
no	74.09	58.5	18.09	20.34	74.09	20.34	4.77
no	92.69	129.85	65	23.32	129.85	65	3.69
no	29.53	33	9.4	11.37	33	11.37	3.88
no	43.15	28.37	18.11	22.46	43.15	22.46	6.3
no	7.56	7.37	17.31	7.35	7.56	17.31	3.25
no	34.4	39.97	28.35	29.09	39.97	29.09	3.93
former	25.47	20.6	14.69	13.75	25.47	14.69	2.98
no	17.5	28.69	16.57	16.56	28.69	16.57	4.17
former	42.03	62.75	30.43	34.96	62.75	34.96	5.48
former	47.88	26.28	22.11	24.44	47.88	24.44	3.56
no	17.5	10.56	15.72	9.93	17.5	15.72	2.4
no	20	14.94	13.71	15.5	20	15.5	3
former	31.54	32.35	32.78	28.21	32.35	32.78	3.39
former	70.91	85.88	40.03	39.81	85.88	40.03	3.6
former	32.46	34.03	20.22	22.88	34.03	22.88	2.67
former	11.07	16.31	30.44	30.63	16.31	30.63	3.4
no	39.07	42.16	8.24	23.4	42.16	23.4	3.99
smoker	38.84	33.62	24.28	26.72	38.84	26.72	4.99
no	41.84	36.34	30.78	33.16	41.84	33.16	3.67
former	34.38	35.01	28.19	24.13	35.01	28.19	2.86
no	70.41	69.59	33.06	48.5	70.41	48.5	3.21
no	50.69	46.85	22.09	31.93	50.69	31.93	3.19
smoker	22.35	26.95	9.63	22.6	26.95	22.6	3.63
no	45.22	26.18	18.19	30.16	45.22	30.16	2.57
former	37.88	34.5	27.37	25.75	37.88	27.37	3.05
no	17.84	12.53	12.6	19.13	17.84	19.13	2.8
no	99.68	131.16	45.31	68.1	131.16	68.1	3.24
no	38.19	38.6	25.09	22.87	38.6	25.09	3.14
no	47.59	16.75	19.7	18.97	47.59	19.7	3.23
no	35.25	30.38	24.18	20.16	35.25	24.18	2.9
no	15.93	19.63	16.81	17.38	19.63	17.38	3.24
no	58.03	65.69	27.44	26.24	65.69	27.44	2.92
no	47.78	58.75	28.82	32.15	58.75	32.15	3.34
no	18.09	18.66	15.5	18.21	18.66	18.21	3.84
former	53.78	53.22	21.91	27.78	53.78	27.78	2.86
no	41.91	36.5	19.16	31.29	41.91	31.29	3.75
no	26.44	34	20.4	13.91	34	20.4	3.07
no	39.75	45.53	19.28	23.44	45.53	23.44	5.68
no	25.4	48.41	17.19	19	48.41	19	3.62
no	56.12	40.37	54.84	58.75	56.12	40.37	2.54
former	13.88	18.65	10.49	13.35	18.65	13.35	3.07
former	38.74	38.48	18.1	19.77	38.74	19.77	2.88
no	28.12	36.41	20.33	21.05	36.41	21.05	3.22
former	52.94	57.37	29.68	40.31	57.37	40.31	3.66
no	16.97	46.62	28.97	31.22	46.62	31.22	3.76
no	13.32	25.06	8.81	12.53	25.06	12.53	3.67
no	52.9	50.16	17.63	28.97	52.9	28.97	4.36
no	22.71	16.6	18.28	13.87	22.71	18.28	2.12
former	11.9	9.03	6.53	6.91	11.9	6.91	4.14
former	32.44	26.44	26.93	24.56	32.44	26.93	2.26
smoker	24.36	19.83	9.12	15.04	24.36	15.04	3.19
former	20.29	31.57	16.68	15.32	31.57	16.68	2.4
former	15.2	52.09	30.24	34.92	52.09	34.92	2.15

no	29.46	43.83	31.45	36.38	43.89	36.38	3.62
former	24.49	47.2	30.42	32.7	47.2	32.7	2.52
no	37.48	67.13	25.25	25.86	67.13	25.86	5.27
former	50.58	47.7	18.39	20.53	50.58	20.53	2.57
no	33.4	32.23	23.46	23.66	33.4	23.66	3.46
no	17.11	15.55	10.17	13.6	17.11	13.6	3.14
no	62.02	40.75	26.73	33.04	62.02	33.04	4.87
no	59.03	44.43	35.28	36.89	59.03	36.89	3.19
no	22.86	29.32	14.9	16.4	29.32	16.4	3.51
no	53.34	46.04	32.75	30.54	53.34	32.75	3.23
former	55.6	56.64	26.96	25.99	56.64	26.96	4.2
no	26.75	47.2	32.7	36.19	47.2	36.19	3.7
no	27.32	24.64	26.74	25.24	27.32	26.74	3.3
no	86.28	94.86	42.29	44.72	94.86	44.72	3.32
no	33.27	45.02	19.1	28.64	45.02	28.64	3.59
no	50.05	73.23	30.68	31.45	73.23	31.45	5.85
no	21.59	17.69	9.74	12.34	21.59	12.34	2.95
no	38.25	35.2	21.19	24.81	38.25	24.81	3.88
no	33.67	33.96	15.98	21.42	33.96	21.42	3.35
smoker	25.65	24.96	17.55	21.83	25.65	21.83	2.3
former	39	37.21	26.68	28.58	39	28.58	2.38
no	15.22	19.86	16	14.8	19.86	16	2.59
no	52.46	64.22	21.31	25.32	64.22	25.32	5.4
no	49.51	47.5	18.77	27.17	49.51	27.17	3.12
no	28.08	39.67	22.26	29.85	39.67	29.85	3.42
smoker	37.18	34.38	32.7	36.99	37.18	36.99	3.01
no	31.12	42.54	29.18	38.54	42.54	38.54	2.65
former	16.36	15.81	25.63	20.12	16.36	25.63	3.72
no	48.99	77.01	35.54	35.74	77.01	35.74	4.22
no	11.68	9.83	11.11	9.49	11.68	11.11	3.64
no	35.79	38.03	15.25	16.33	38.03	16.33	3.47
smoker	38.05	45.89	22.25	24.898	45.89	24.89	3.73
no	70.89	84.89	27.44	34.51	84.89	34.51	3.88
no	80	113.44	40.18	66.27	113.44	66.27	4.23
former	11	13.82	10.06	9.11	13.82	10.06	2.25
no	47.63	43.67	17.13	26.99	47.63	26.99	2.53
former	38.77	39.02	22.18	25.93	39.02	25.93	2.63
former	39	62.23	37.51	35.85	62.23	37.51	4.13
former	30.66	27.26	22.36	20.21	30.66	22.36	2.43
former	26.15	31.95	25.33	24.98	31.95	25.33	3.59
no	58.67	59.77	75.19	50.38	59.77	75.19	2.44
no	69.04	76.7	34.47	37.88	76.7	37.88	3.9
no	41.51	88.69	38.4	39.09	88.69	39.09	2.62
former	34.99	37.59	25.34	28.72	37.59	28.72	2.56
former	28.66	40.82	32.13	28.24	40.82	32.13	3.71
no	59.71	25.87	24.92	15.83	59.71	24.92	2.56
no	38.06	29.47	20.07	19.46	38.06	20.07	3.28
no	86.28	83.54	44.74	48.35	86.28	48.35	5.69
no	23.24	31.07	13.57	14.39	31.07	14.39	3.42
former	30.18	22.8	20.62	20.05	30.18	20.62	4.56
no	14.84	17.12	14.48	15.06	17.12	15.06	2.73
former	48.29	55.09	36.92	50.51	55.09	50.51	3.29
former	69.04	78.53	18.81	21.36	78.53	21.36	5.77
former	17.43	19.61	25.6	23	19.61	25.6	2.6
no	53.68	49.71	23.06	27.94	53.68	27.94	4.5
no	34.16	41.88	26.91	27.5	41.88	27.5	3.71
no	55.28	46.84	18.81	17.38	55.28	18.81	4.69
no	92.47	97.03	25.16	26.62	97.03	26.62	3.88
former	18.44	21.01	14.53	14.81	21.01	14.81	2.69

no	57.61	77.64	29.12	34.02	77.64	34.02	3.1
no	23.25	31.86	19.98	32.24	31.86	32.24	5.43
former	29.2	28.86	19.99	20.24	29.2	20.24	3.37
smoker	39.64	38.23	20.05	29.29	39.64	29.29	2.49
no	50.02	41.92	24.84	26.59	50.02	26.59	3.72
former	36.32	37.05	20.48	18.14	37.05	20.48	3.57
no	8.32	46.36	17.55	26.47	46.36	26.47	3.98
smoker	12.2	7.19	12.97	6.91	12.2	12.97	2.95
former	13.31	10.89	13.53	11.43	13.31	13.53	2.67
former	11.08	8.72	7.25	9.44	11.08	9.44	2.8
smoker	25.68	35.9	25.98	25.36	35.9	25.98	3.29
no	63.86	72.07	16.01	15.56	72.07	16.01	3.95
no	45.97	61.28	18.41	24.01	61.28	24.01	5.05
former	32.25	32.85	21.02	22.95	32.85	22.95	3.1
no	22.71	26.76	23.33	23.5	26.76	23.5	4.63
former	37.41	41.38	26.69	23.32	41.38	26.69	3.69
no	43.68	45.64	35.56	28.17	45.64	35.56	4.12
former	48.27	47.69	35.87	33.63	48.27	35.87	2.69
no	33.79	26.75	11.75	13.22	33.79	13.22	3.85
former	36.87	34.87	25.82	25.46	36.87	25.82	3
former	40.63	46.79	20.89	27.98	46.79	27.98	2.4
no	41.41	52.19	23.59	28.5	52.19	28.5	2.75
no	38.06	47.17	27.87	29.76	47.17	29.76	3.39
no	54.41	65.28	25.45	28.17	65.28	28.17	3.91
no	43.4	74.06	24.66	32.21	74.06	32.21	3.03
no	31.14	34.37	40.31	31.36	34.37	40.31	2.88
no	40.95	39.29	17.87	20.78	40.95	20.78	2.6
no	6.85	13.71	8.17	10.86	13.71	10.86	4.26
no	30.96	37.75	12.95	18.04	37.75	18.04	3.1
no	14.15	11.57	9.42	22.86	14.15	22.86	2.59
no	19.63	24.84	20.48	21.77	24.84	21.77	3.32
no	83.02	108.13	40.66	44.54	108.13	44.54	4.55
no	17.74	11.87	9.45	8.95	17.74	9.45	3.09
former	27.34	31.61	23.37	18	31.61	23.37	2.66
smoker	27.43	35.83	19.33	8.93	35.83	19.33	2.82
no	57.49	59.64	28.67	32.43	59.64	32.43	3.13
no	29.86	42.94	29.38	30.67	42.94	30.67	4.11
no	13.84	17.25	15.01	19.79	17.25	19.79	5.92
no	12.29	9.49	7.69	9	12.29	9	2.73
former	15.03	25.99	18.23	19.7	25.99	19.7	2.8
no	25.87	27.71	21.53	21.56	27.71	21.56	3.28
no	16.11	17.42	16.31	16.03	17.42	16.31	3.2
no	40.53	69.18	29.66	26.46	69.18	29.66	3.52
no	61.61	76.13	20.82	17.63	76.13	20.82	4.58
no	22.12	23.76	39.81	41.71	23.76	41.71	3.49
no	58.19	76.96	36.29	56.03	76.96	56.03	3.7
former	30.42	28.63	29.09	28.11	30.42	29.09	2.49
former	21.61	25.94	21.36	15.19	25.94	21.36	2.36
no	28.47	73.76	28.55	22.81	73.76	28.55	2.89
no	59.23	52.46	41.63	51.89	59.23	51.89	2.36
former	17.76	30.35	18.65	30.92	30.35	30.92	2.61
no	29.29	35.41	18.77	25.09	35.41	25.09	2.67
former	52.85	53.92	37.78	33.52	53.92	33.52	2.84
no	93.65	75.03	53.65	60.31	93.65	60.31	3.3
no	62.14	66.12	25.24	38.03	66.12	38.03	4.25
no	53.48	58.5	34.72	41.44	58.5	41.44	3.58
no	47.95	47.04	43.23	35.05	47.95	43.23	3.77
no	30.4	45.27	25.95	36.83	45.27	36.83	3.39
no	43.85	48.15	23.2	22.73	48.15	23.2	3.1

no	39.79	54.25	28.22	28.98	54.25	28.98	3.08
no	68.29	76.65	15.72	25.62	76.65	25.62	5.04
no	75.64	72.72	20.05	27.82	75.64	27.82	5.86
no	70.95	101.81	29.3	30.83	101.81	30.83	5.38
no	28.83	40.94	41.88	26.3	40.94	41.88	2.81
no	51.45	53.75	29.75	33.07	53.75	33.07	3.4
no	57.46	69.55	34.58	38.3	69.55	38.3	3.25
no	60.47	57.46	30.98	39.67	60.47	39.67	3.34
no	66.19	69.95	56.35	51.25	69.95	56.35	3.62
no	12.26	31.7	26.39	23.8	31.7	26.39	3.77
smoker	12.85	11.15	9.88	10	12.85	10	3.3
former	36.26	28.91	21.04	28.96	36.26	28.96	2.2
former	41.5	42.6	12.46	14.34	42.6	14.34	3.92
no	26.72	21.95	14.93	19.65	26.72	19.65	3.04
no	29.68	34.72	22.85	25.46	34.72	25.46	2.21
no	26.9	37.02	19.85	23.42	37.02	23.42	3.67
no	25.56	60.85	39.69	34.85	60.85	39.69	3.81
no	48.66	68.75	32.16	32.93	68.75	32.93	5.24
smoker	41.83	45.53	26.75	30.06	45.53	30.06	4.09
no	23.73	30.25	19.01	24.37	30.25	24.37	3.48
no	11.35	15.11	12.09	7.63	15.11	12.09	3.36
former	30.15	32.21	25.37	36.09	32.21	36.09	3.22
no	41.33	19.8	18.82	32.65	41.33	32.65	2.86
no	83.77	82.93	46.07	45.73	83.77	46.07	4.7
former	11.02	20.4	20.19	25.6	20.4	25.6	4.7
no	43.37	43.93	11.63	17.05	43.93	17.05	2.46
former	29.96	31.7	17.89	23.47	31.7	23.47	3.58
no	14.94	21.75	19.64	21.66	21.75	21.66	3.12
no	32.96	46.79	31.15	25.54	46.79	31.15	3.33
no	32.55	37.12	16.13	12.46	37.12	16.13	2.94
former	7.6	4.28	6.73	13.06	7.6	13.06	2.5
smoker	19.04	23.69	18.8	19.66	23.69	19.66	2.35
former	20.97	25.93	9.67	18.28	25.93	18.28	2.81
former	22.83	48.3	37.39	43.25	48.3	43.25	3.29
smoker	32.68	20.75	16	29.48	32.68	29.48	2.55
no	45.56	63.8	28.18	34.23	63.8	34.23	4.35
no	104.09	113.45	32.23	44.18	113.45	44.18	4.51
no	33.85	46.46	24.42	24.01	46.46	24.42	4.2
no	70.58	44.57	31.93	36.83	70.58	36.83	4.5
no	12.31	10.74	11.61	11.5	12.31	11.61	2.94
no	24.1	23.39	12.46	10.83	24.1	12.46	4.14
no	49.47	56.19	37.08	47.73	56.19	47.73	3.39
no	25.46	28.03	20.82	24.32	28.03	24.32	3.11
former	15.77	17.2	15.05	9.73	17.2	15.05	2.5
no	21.34	19.55	23.71	21.29	21.34	23.71	4.96
no	39.12	32.45	20.62	21.84	39.12	21.84	3.16
no	57.99	63.8	45.45	48.61	63.8	48.61	2.9
no	39.34	40.51	36.62	35.59	40.51	36.62	3.04
no	27.5	31.6	6.76	9.9	31.6	9.9	2.88
former	18.28	15.68	14.98	21.12	18.28	21.12	3.39
former	25.95	28.44	21.51	21.63	28.44	21.63	2.59
former	33.29	34.11	3.78	8.76	34.11	8.76	3.26
smoker	31.63	36.94	28.3	29.74	36.94	29.74	2.28
no	19.5	25.5	25.62	33.62	25.5	33.62	3.25
no	34.74	45.06	27.91	32.23	45.06	32.23	2.82
no	15.09	26.42	17.95	21.39	26.42	21.39	2.92
no	79.02	90.69	37.85	46.4	90.69	46.4	4.2
no	56.06	77.04	26.89	39.8	77.04	39.8	5.83

FVC N	FVC Pre	VC Pre %Pre	FCV After	FCV after %Prev	FVC %Des	FEV1 Prev
1.86	2.56	101.2	2.62	103.6	2.3	1.93
2.62	2.22	63.7	2.23	64	0.5	2.78
1.86	2.08	82.2	2.23	88.1	7.2	2.03
2.83	3.08	82.9	3.3	89.7	8.2	3.2
2.06	2.1	76.8	2.33	85.2	11	2.24
1.84	2	79.7	2.4	95.6	20	1.94
3.41	2.11	50.2	2.09	49.8	0.9	3.57
2.8	1.53	44.3	1.61	46.7	5.2	3.01
2.31	2.18	71	2.18	71	0	2.46
2.04	2.28	83.8	2.22	81.6	-2.6	2.22
2.45	1.73	51.8	NA	NA	NA	2.55
1.96	2.35	90.4	2.29	88.1	-2.6	2.14
3.07	4.28	107.3	3.97	99.5	-7.2	3.4
2.3	2.49	81.4	2.51	82	0.8	2.52
2.44	3.2	100.6	3.28	103.1	2.5	2.7
2.82	3.33	86.7	3.11	81	-6.6	3.12
1.95	2.48	91.2	NA	NA	NA	2.15
2.69	2.29	65.4	2.37	67.7	3.5	2.98
1.66	1.64	72.6	1.93	85.4	17.7	1.77
3.78	4.42	94.8	4.46	95.7	0.9	3.95
2.47	2.72	80.7	2.73	81	0.4	2.65
2.65	1.18	34.2	1.45	42	22.9	3.01
2.15	2.94	105	2.93	104.6	0.3	2.42
4.23	4.91	94.2	4.83	92.7	-1.6	4.36
3.53	3.06	70.3	3.19	73.3	4.2	3.64
1.68	1.66	72.8	1.73	75.9	4.2	1.75
3.48	4.57	96.4	5.03	106.1	10.1	3.9
1.7	2.47	109.8	NA	NA	NA	1.81
2.27	3.8	1123	3.84	124.3	1.1	2.44
2.63	2.59	74	2.9	82.9	12	2.88
2.41	2.37	73.8	2.72	84.7	14.8	2.58
4.35	4.15	77.4	4.41	82.3	6.3	4.41
2.27	2.4	79.5	2.58	85.4	7.5	2.54
2.25	2.47	84.3	2.44	83.3	-1.2	2.57
2.29	2.13	71.7	2.18	73.4	2.3	2.56
2.43	2.58	81.6	2.5	79	-3.1	2.74
2.5	2.75	82.6	NA	NA	NA	2.77
1.58	1.57	71.4	1.55	70.5	-1.3	1.6
2.25	1.83	61.2	NA	NA	NA	2.39
2.63	1.88	52.5	1.91	53.4	1.6	2.78
1.76	1.8	76.8	1.95	83.2	8.3	1.88
3.26	1.55	34.9	1.85	41.7	19.4	3.6
1.81	1.91	79.3	NA	NA	NA	2.01
2.09	2.46	88.5	NA	NA	NA	2.27
2.55	2.85	85.8	2.73	82.2	-4.2	2.83
2.02	2.13	79.2	2.38	88.5	11.7	2.2
2.34	2.45	78.8	2.51	80.7	2.4	2.63
2.5	2.49	76.6	3.04	93.5	22.1	2.75
1.93	2.29	85.3	2.14	79.7	-6.6	2.13
2.25	2.93	95.8	2.96	96.7	1	2.43
1.7	2.26	100	2.3	101.8	1.8	1.84
2.62	3.34	98.2	3.41	100.3	2.1	2.92
2.01	1.53	57.3	1.48	55.4	-3.3	2.13
1.8	1.37	54.8	1.37	54.8	0	2
2.28	4.4	141.9	4.38	141.3	0.5	2.51
2.91	2.07	52.3	2.02	51	-2.4	3.14
2.23	2.83	95.6	2.93	99	3.5	2.43
1.79	2.17	91.2	2.24	94.1	3.2	1.97

1.79	1.99	83.6	1.94	81.5	-2.5	1.94
2.91	2.79	73.8	2.8	74.1	0.4	3.28
1.78	1.57	66.5	1.7	72	8.3	1.95
2.26	3.04	103.4	2.93	99.7	-3.6	2.55
2.82	3.88	103.5	4.09	109.1	5.4	3.06
4.43	4.99	91.3	4.91	89.9	-1.6	4.56
3.87	3.97	83.2	4.08	85.5	2.8	3.95
2.71	3.62	98.1	3.58	97	-1.1	2.95
2.85	3.6	92.8	3.62	93.3	0.6	3.07
5.11	5.01	79.5	4.82	76.5	-3.8	5.26
2.45	2.1	64.6	2.52	77.5	20	2.72
3.19	2.86	72.8	2.89	73.6	1	3.38
2.24	1.2	40.3	1.38	46.3	15	2.45
3.06	3.59	86.1	3.92	94	9.2	3.42
4.02	3.92	71.5	NA	NA	NA	4.31
2.62	3.39	95.2	3.07	86.2	-9.4	2.81
1.81	1.31	54.6	1.36	56.7	3.8	1.93
2.26	1.83	61	2.43	81	32.8	2.52
2.55	2.17	63.9	2.09	61.6	-3.7	2.71
2.64	3.55	98.6	3.61	100.3	1.7	2.86
2.01	1.52	56.9	1.61	60.3	5.9	2.18
2.5	3.56	104.7	NA	NA	NA	2.7
2.93	3.28	82.3	NA	NA	NA	3.24
4.05	4.91	98.3	4.63	92.7	-5.7	4.12
2.83	3.34	91	3.29	89.6	-1.5	3.14
2.1	3.17	110.8	3.34	116.8	5.4	2.27
2.47	3.01	93.8	2.96	92.2	-1.7	2.72
2.4	2.84	89	2.81	88.1	-1.1	2.7
2.8	3.33	91.6	3.14	86.4	-5.7	3.08
1.94	1.69	65.78	1.69	65.8	0	2.12
2.29	1.99	65.2	2.07	67.9	4	2.47
2.1	1.78	63.6	1.85	66.1	3.9	2.29
2.44	4.04	124.7	4.1	126.5	1.5	2.67
2.41	2.56	81.5	2.91	92.7	13.7	2.69
2.37	2.37	73.4	2.3	71.2	-3	2.57
2.18	2.04	70.3	2.09	72.1	2.5	2.4
2.44	2.59	79.9	2.64	81.5	1.9	2.73
2.2	2.84	97.3	2.87	98.3	1.1	2.5
2.57	3.39	101.5	3.34	100	-1.5	2.9
2.95	3.9	101.6	3.86	100.5	-1	3.23
2.15	1.66	72.5	1.69	73.8	1.8	2.29
2.75	4.21	112.3	4.12	109.9	-2.1	3.03
2.31	1.85	60.3	1.91	62.2	3.2	2.46
4.6	2.83	49.8	2.79	49.1	-1.4	4.77
2.72	3.04	84	2.95	81.5	-3	2.98
1.82	2.68	105.5	2.75	108.3	2.6	1.94
2.31	1.68	54.7	1.29	42	-23.2	2.48
2.17	1.61	55.9	1.84	63.9	14.3	2.3
2.48	2.68	83.2	3.17	98.4	18.3	2.74
2.69	2.1	57.3	2.94	80.2	40	2.85
2.89	2.52	67	NA	NA	NA	3.24
2.83	2.18	59.4	2.21	60.2	1.4	3.14
3.54	2.92	67	3.15	72.2	7.9	3.66
1.6	1.37	64.6	NA	NA	NA	1.72
3.04	1.37	33.1	1.44	34.8	5.1	3.41
1.62	1.48	65.5	1.63	72.1	10.1	1.78
2.34	3.34	104.8	3.37	105.7	0.9	2.57
1.73	1.82	75.8	1.74	72.5	-9.4	1.98
1.54	2.05	95.3	2.19	101.9	6.8	1.45

2.72	2.97	82	2.96	81.8	0.3	3.01
1.85	3.26	129.4	3.28	130.2	0.6	1.99
4.28	4.08	77.4	4.2	79.7	2.9	4.42
1.93	2.03	79	1.96	76.3	-3.4	2.05
2.66	3.23	93.4	3.18	91.9	-1.5	2.92
2.41	3.15	100.4	3.07	97.9	-2.5	2.72
3.95	4.8	98.6	4.59	94.3	-4.4	4.08
2.4	3.32	104.1	NA	NA	NA	2.62
2.84	1.7	48.4	1.83	52.1	7.6	3.05
2.43	2.65	82.1	2.65	82.1	0	2.63
3.08	3.31	78.8	3.34	79.5	0.9	3.45
2.85	3.74	101.1	3.58	96.8	-4.3	3.16
2.48	3.65	110.6	NA	NA	NA	2.67
2.44	2.97	89.5	2.87	86.4	-3.4	2.66
2.76	3.3	91.9	3.31	92.2	0.3	3.02
4.75	4.53	77.4	NA	NA	NA	4.85
2.22	1.78	60.3	1.87	63.4	5.1	2.41
2.85	2.78	71.6	2.87	74	3.2	3.07
2.46	2.91	86.8	3.28	97.9	12.7	2.59
1.73	0.92	40	NA	NA	NA	1.85
1.79	2.35	98.8	2.37	99.6	0.9	1.97
1.95	0.95	36.7	0.85	32.8	-10.5	2.17
4.38	3.41	63.1	3.42	63.3	0.3	4.48
2.35	2.57	82.4	NA	NA	NA	2.57
2.57	2.27	66.4	NA	NA	NA	2.86
2.27	2.42	80.3	NA	NA	NA	2.48
1.91	1.63	61.5	1.77	66.8	8.6	2.09
2.73	3.11	83.6	3.48	93.5	11.9	2.98
3.09	5.15	122.2	5.05	119.8	-1.9	3.43
2.74	3.44	94.6	NA	NA	NA	3
2.67	2.14	61.7	2.24	64.6	4.7	3.03
2.87	3.63	97.4	NA	NA	NA	3.12
2.85	3.92	101.1	NA	NA	NA	3.17
3.1	4.46	105.6	NA	NA	NA	3.48
1.62	1.6	71.1	NA	NA	NA	1.88
1.82	2.38	94	2.51	99.1	5.5	1.99
1.98	1.99	75.7	2.05	78	3	2.15
3.03	3.56	86.2	3.54	85.7	0.6	3.25
1.83	2.15	88.5	2.01	82.7	-6.5	2
2.64	3.36	93.6	3.28	91.4	-2.4	2.84
1.79	2.34	95.9	2.62	107.4	12	1.92
2.93	2.86	73.3	3.1	79.5	8.4	3.15
1.97	2.45	93.4	NA	NA	NA	2.13
1.84	1.69	66	NA	NA	NA	2.05
2.72	3.56	96	3.73	100.5	4.8	2.94
1.84	2.35	91.8	2.29	89.5	-2.6	2.03
2.47	2.23	68	2.36	72	5.8	2.6
4.62	3.99	70.1	4.06	71.4	1.8	4.75
2.57	2.85	83.3	2.78	81.3	-2.5	2.81
3.35	3.98	87.3	NA	NA	NA	3.62
2.05	2.51	91.9	NA	NA	NA	2.21
2.41	2.8	85.1	2.97	90.3	6.1	2.61
4.68	4.98	86.3	NA	NA	NA	4.8
1.95	2.41	92.7	NA	NA	NA	2.09
3.3	5.05	112.3	NA	NA	NA	3.57
2.85	3.21	86.6	NA	NA	NA	3.13
3.8	4.81	102.6	NA	NA	NA	3.88
3.15	3.39	87.4	3.38	87.1	0.3	3.29
1.97	2.19	81.4	2.34	87	6.8	2.1

2.38	2.48	80	2.59	83.5	44	2.66
3.99	4.63	85.2	NA	NA	NA	4.42
2.59	2.99	88.7	2.86	84.9	-4.3	2.85
1.88	1.96	78.6	2.03	81.4	3.6	2
2.86	3.52	95.6	NA	NA	NA	3.18
2.68	2.62	73.4	NA	NA	NA	2.93
3.06	3.44	86.4	NA	NA	NA	3.4
2.22	2.56	86.8	NA	NA	NA	2.48
2.01	1.77	66.4	2.06	77.2	16.4	2.16
2.11	2.06	73.6	2.19	78.2	6.3	2.31
2.47	2.3	69.9	2.41	73.3	4.8	2.63
3.21	3.12	79	3.47	87.8	11.2	3.39
3.71	3.89	77	3.84	76	-1.3	4.15
2.33	2.17	70	2.27	73.2	4.6	2.45
3.76	3.32	71.7	NA	NA	NA	3.84
2.71	2.58	69.9	2.76	74.8	7	2.95
3.02	2.9	70.4	2.97	72.1	2.4	3.3
1.93	1.55	57.6	1.75	65.1	12.9	2.03
2.96	3.19	83	NA	NA	NA	3.23
2.2	3.13	104.3	3.19	106.3	1.9	2.38
1.81	1.82	75.7	1.81	75.3	0.5	1.93
2.07	2.45	89.2	NA	NA	NA	2.28
2.49	3.43	101.2	NA	NA	NA	2.7
2.87	4.21	107.7	4.28	109.5	1.7	3.22
2.28	3.48	114.8	NA	NA	NA	2.5
2.17	2.87	99.7	NA	NA	NA	2.3
2	2.36	90.8	NA	NA	NA	2.32
3.13	2.4	56.3	NA	NA	NA	3.43
2.3.9	2.45	78.9	NA	NA	NA	2.74
1.95	2.08	80.3	NA	NA	NA	2.12
2.5	2.71	81.5	2.47	74.3	-8.9	2.72
3.69	3.97	87.2	NA	NA	NA	3.8
2.32	2.56	83	2.4	77.8	-6.2	2.53
1.95	1.81	68.1	1.83	68.9	1.1	2.07
2.07	1.39	49.3	1.47	52.1	5.8	2.18
2.35	3.79	121.2	3.76	120.2	0.8	2.51
3.16	3.1	75.4	2.88	70.1	-7.1	3.48
4.8	3.98	67.2	3.94	66.6	-1	4.92
2.05	1.02	37.3	NA	NA	NA	2.18
2.06	2.08	74.2	2.07	73.8	0.5	2.21
2.47	2.19	66.8	2.36	72	7.8	2.65
2.4	2.16	67.6	NA	NA	NA	2.59
2.71	3.31	94	NA	NA	NA	3.05
3.71	4.32	94.4	NA	NA	NA	3.81
2.69	3.47	99.4	NA	NA	NA	3.04
2.79	4.11	111.1	NA	NA	NA	3.08
1.88	2.28	91.6	2.38	95.6	4.4	2
1.73	2.36	100	NA	NA	NA	1.78
2.17	2.53	87.5	NA	NA	NA	2.38
1.73	2.36	100	NA	NA	NA	1.78
1.96	2.28	87.4	2	76.6	-12.3	2.08
2.01	2.15	80.5	NA	NA	NA	2.18
2.09	2.48	87.3	2.87	101.1	15.7	2.19
2.54	3.23	97.8	NA	NA	NA	2.86
3.27	4.89	115.1	NA	NA	NA	3.58
2.69	3.36	93.9	NA	NA	NA	2.97
2.9	3.71	98.4	NA	NA	NA	3.21
2.55	3.19	94.2	NA	NA	NA	2.82
2.38	2.36	76.1	2.57	82.9	8.9	2.66

2.31	3.74	121.4	3.76	122.1	0.5	2.59
4.09	5.79	114.8	NA	NA	NA	4.23
4.75	5.05	86.2	NA	NA	NA	4.87
4.36	5.96	110.8	5.88	109.3	-1.3	4.48
2.11	2.46	87.6	2.47	88	0.4	2.31
2.62	3.21	94.3	3.29	96.7	2.5	2.92
2.5	3.8	117	NA	NA	NA	2.83
2.57	3.25	97.3	NA	NA	NA	2.82
2.78	3.28	90.6	NA	NA	NA	3.12
2.9	2.6	69	2.62	69.5	0.8	3.23
2.48	1.85	56.1	1.81	54.8	-2.2	2.67
1.61	1.73	78.6	1.8	81.8	4	1.69
2.88	2.73	69.6	2.97	75.7	8.8	3.07
2.29	1.67	54.9	1.69	55.6	1.2	2.45
1.59	1.36	58.8	1.68	76	29.2	1.5
2.765	3.83	104.3	3.96	107.9	3.4	3.04
2.93	3.62	95.1	NA	NA	NA	3.24
3.85	4.3	82.1	NA	NA	NA	4.2
3	3.49	85.3	3.39	82.9	-2.9	3.28
2.68	3.33	95.7	NA	NA	NA	2.96
2.53	3.75	111.6	NA	NA	NA	2.73
2.36	3.94	122.4	4.01	124.5	1.8	2.6
2.15	2.41	84.3	2.62	91.6	8.7	2.34
3.45	4.24	90.2	4.33	92.1	2.1	3.83
3.45	4.11	87.4	4.49	95.5	9.2	3.85
1.76	1.9	77.3	1.8	73.3	-5.3	1.93
2.69	2.65	74	NA	NA	NA	2.92
2.35	2.6	83.3	NA	NA	NA	2.62
2.5	2.05	61.6	2.35	70.6	14.6	2.74
2.21	2.03	69	2.09	71.1	3	2.41
1.88	0.99	39.6	1.15	46	16.2	2.08
1.76	1.5	63.8	1.53	65.1	2	1.93
2.12	1.24	44.1	1.43	50.8	15.3	2.31
2.47	2.59	78.7	2.95	89.7	13.9	2.71
1.92	2.39	93.7	2.43	95.3	1.7	2.08
3.2	3.23	74.3	NA	NA	NA	3.54
3.66	4.51	100	NA	NA	NA	3.8
3.08	3.34	79.5	NA	NA	NA	3.35
3.3	4.08	90.7	NA	NA	NA	3.66
2.21	1.8	61.2	NA	NA	NA	2.49
3.04	4.72	114	NA	NA	NA	3.39
2.55	3.64	107.4	NA	NA	NA	2.82
2.28	2.76	88.8	NA	NA	NA	2.53
1.88	2.1	84	2	80	-4.8	2.02
3.64	4.14	83.4	NA	NA	NA	4.09
2.32	2.73	86.5	NA	NA	NA	2.54
2.18	2.83	97.6	3.02	104.1	6.7	2.43
2.29	2.98	98.1	2.91	95.8	-2.3	2.45
2.17	1.9	66	1.97	68.4	3.7	2.36
2.55	2.15	63.4	NA	NA	NA	2.74
1.86	1.56	60.2	1.85	71.4	18.6	2.09
2.39	2.49	76.4	2.95	90.5	18.5	2.6
1.71	1.46	64	1.68	73.7	15.1	1.86
2.44	3.24	99.7	3.13	96.3	-3.4	2.7
2.12	2.24	79.5	NA	NA	NA	2.31
2.2	1.95	66.8	NA	NA	NA	2.37
3.08	4.63	110.2	NA	NA	NA	3.45
4.73	5.07	86.9	NA	NA	NA	4.86

FEV1 N	FEV1 Pre	V1 Pre %P	FEV1 After	√1 after %F	FEV1 %Des	FEV1/FCV Prev
1.49	2.56	105.2	2.03	105.2	0	78.08
2.16	2.22	67.3	1.93	69.5	3.2	77.7
1.57	2.08	35.5	0.8	39.4	11.1	79.86
2.25	3.08	57.8	2.42	75.6	30.8	88.07
1.74	2.1	61.2	1.52	67.9	10.9	81.6
1.5	2	58.8	1.33	68.6	16.7	78.6
2.82	2.11	37.6	1.3	36.5	-3	83.96
2.38	1.53	30.2	0.95	31.6	4.4	84.59
1.92	2.18	70.3	1.78	72.4	2.9	79.17
1.73	2.28	86	1.99	89.6	4.2	81.41
1.96	1.73	35.7	NA	NA	NA	77.66
1.67	2.35	68.2	1.53	71.5	4.8	82.16
2.39	4.28	109.1	3.58	105.3	-3.5	85.46
1.96	2.49	76.2	1.99	79	3.6	81.81
1.9	3.2	84.4	2.33	86.3	2.2	85.21
2.41	3.33	78.5	2.52	80.8	2.9	80.91
1.56	2.48	93	NA	NA	NA	78.96
2.1	2.29	45.9	1.4	46.9	2.2	85.42
1.36	1.64	45.8	1.03	58.2	27.2	78.92
3.12	4.42	85.1	3.63	91.9	8	84.38
2.05	2.72	60.8	1.7	64.2	5.6	79.23
2.11	1.18	28.2	0.91	30.2	7.1	88.24
1.7	2.94	111.2	2.74	113.2	1.9	87.07
3.45	4.91	89.2	3.9	89.4	0.3	83.96
2.88	3.06	57.7	2.1	57.7	0	83.01
1.35	1.66	41.7	0.87	49.7	19.2	78.39
3.01	4.57	63.6	2.71	69.5	9.3	82.28
1.41	2.47	107.7	NA	NA	NA	81
1.89	3.8	79.9	2.01	82.4	3.1	79.44
2.24	2.59	53.8	1.9	66	22.6	81.28
2.01	2.37	47.3	1.45	56.2	18.9	79.37
3.49	4.15	77.3	3.77	85.8	10.6	82.7
1.98	2.4	79.9	2.2	86.6	8.4	84.07
1.81	2.47	89.1	2.32	90.3	1.3	88.58
1.8	2.13	75.8	2.07	80.9	6.7	86.33
1.92	2.58	63.6	1.74	63.6	0	87.09
2.16	2.16	78	NA	NA	NA	82.77
1.16	1.57	76.2	1.22	76.2	0	77.61
1.86	1.83	41	NA	NA	NA	78.98
2.15	1.88	38.5	1.12	40.3	1.6	78.5
1.47	1.8	71.2	1.39	73.9	3.7	80.63
2.78	1.55	23.9	1.14	31.7	32.6	81.23
1.57	1.91	60.7	NA	NA	NA	83.46
1.77	2.46	80.2	NA	NA	NA	81.41
1.99	2.85	83	2.37	83.7	0.9	85.41
1.72	2.13	55.5	1.59	72.3	30.3	81.79
2.05	2.45	57.8	1.53	58.2	0.7	84.45
1.93	2.49	65.8	2.21	80.4	22.1	84.65
1.55	2.29	64.2	1.32	61.8	-3.6	79.14
1.88	2.93	90.5	2.31	95.1	5	79.65
1.43	2.26	60.9	1.1	59.8	-1.8	81.75
2.05	3.34	87.3	2.82	96.6	10.6	86.36
1.66	1.53	58.2	1.22	57.3	-1.6	79.52
1.45	1.37	36	0.81	40.5	12.5	79.51
1.93	4.4	113.9	2.94	117.1	2.8	80.39
2.42	2.07	34.7	1.15	36.6	5.5	79.55
1.89	2.83	98.4	2.4	98.8	0.4	81.43
1.54	2.17	75.1	1.49	75.6	0.7	83.08

1.51	1.99	86.6	1.7	87.6	1.2	81.76
2.31	2.79	69.2	2.53	77.1	11.5	87.89
1.52	1.57	57.9	1.21	62.1	7.1	82.89
1.79	3.04	89.4	2.26	88.6	0.9	87.27
2.38	3.88	102.9	3.29	107.5	4.4	79.98
3.61	4.99	74.7	4.16	91.2	22	83.96
3.12	3.97	77.5	3.24	82	5.9	82.7
2.28	3.62	98.3	3.08	104.4	6.2	80.07
2.37	3.6	56	1.86	60.6	8.1	79.55
4.16	5.01	82.5	4.36	82.9	0.5	84.8
2.12	2.1	38.6	1.49	54.8	41.9	83.33
2.67	2.86	58.6	2.08	61.6	5.1	84.48
1.91	1.2	22	0.6	24.5	11.1	81.62
2.64	3.59	76.6	2.98	87.1	13.7	81.75
3.33	3.92	61.9	NA	NA	NA	79.23
2.17	3.39	68	1.92	68.3	0.5	79.34
1.51	1.31	61.7	1.29	66.8	8.4	80.63
1.96	1.83	60.8	1.95	77.5	27.5	83.88
2.11	2.17	64.9	1.7	62.7	-3.4	78.07
2.21	3.55	101.4	2.92	102.1	0.7	79.76
1.7	1.52	28.4	0.68	31.2	9.7	81.6
2.09	3.56	89.6	NA	NA	NA	79.65
2.5	3.28	85.1	NA	NA	NA	81.12
3.26	4.91	97.2	3.95	95.9	-1.2	82.59
2.21	3.34	89.5	2.88	91.7	2.5	86
1.75	3.17	104	2.59	114.1	9.7	79.55
1.91	3.01	85.7	2.4	88.2	3	84.84
2.1	2.84	85.2	2.37	87.8	3	84.65
2.17	3.33	98.3	2.88	93.4	-5	84.87
1.65	1.69	69.8	1.52	71.7	2.7	82.53
1.92	1.99	53.4	1.54	62.3	16.7	80.3
1.78	1.78	55	1.33	58.1	5.6	81.6
2.08	4.04	115.4	3.22	120.6	4.5	81.82
1.89	2.56	57.2	1.84	68.4	19.5	86.15
1.98	2.37	76.3	1.87	72.8	-4.6	79.65
1.87	2.04	73.3	1.83	76.2	4	82.74
2.12	2.59	67.4	1.84	67.4	0	83.9
1.95	1.95	89.6	2.36	94.4	5.4	85.76
2.04	2.88	99.3	2.94	101.4	2.1	87.67
2.27	3.32	102.8	3.47	107.4	4.5	83.75
1.78	1.66	72.5	1.69	73.8	1.8	79.54
2.34	3.66	120.8	3.7	122.1	1.1	80.6
1.92	1.49	60.6	1.59	64.6	6.7	79.17
3.77	2.56	53.7	2.69	56.4	5.1	84.69
2.32	2.03	68.1	2	67.1	-1.5	81.29
1.41	1.43	73.7	1.47	75.8	2.8	78.2
1.93	1.17	47.2	0.97	39.1	-17.1	79.93
1.79	1.01	43.9	1.16	50.4	14.9	79.16
1.92	1.54	56.2	2.11	77	37	85.03
2.2	1.24	43.6	1.44	50.6	16.1	78.5
2.28/	1.7	52.5	NA	NA	NA	86.95
2.21	1.42	45.2	1.53	48.7	7.7	86
2.89	1.57	42.9	1.67	45.6	6.47	83.33
1.34	0.88	51.2	NA	NA	NA	81.56
2.63	0.7	20.5	0.78	22.9	11.4	81.96
1.29	0.65	36.5	0.8	44.9	23.1	79.49
1.99	2.11	82	2.21	85.9	4.7	80.39
1.44	1.52	76.8	1.52	75.8	0	80.44
1.05	1.36	93.8	1.48	102.1	8.8	76.11

2.35	2.31	76.7	2.43	80.7	5.2	82.61
1.53	2.67	134.2	2.7	135.7	1.1	79.23
3.5	3.14	71	3.45	78.1	99	84.17
1.59	1.56	76.1	1.53	74.6	-1.9	79.7
2.05	2.78	95.2	2.8	95.9	0.7	84.29
1.91	1.82	66.9	1.99	73.1	9.3	87.47
3.95	3.76	92.2	3.6	88.2	-4.3	83.64
2.04	2.62	100	NA	NA	NA	81.26
2.41	1.01	33.1	1.09	35.7	7.9	84.48
2.05	1.97	75	1.85	70.4	-6.1	80.32
2.66	2.83	82	2.9	84.1	2.5	81.86
2.22	3.38	107	3.29	104.1	-2.7	85.63
2.08	2.9	108.6	NA	NA	NA	79.76
2.05	2.56	96.2	2.64	99.2	3.1	79.97
2.12	2.88	95.4	2.89	95.7	0.3	83.73
3.83	3.74	77.1	NA	NA	NA	83.54
1.88	1.37	56.8	1.49	61.8	8.8	81.24
2.37	2.49	81.1	2.56	83.4	2.8	79.55
2	1.9	73.4	2.3	88.9	21.1	78.29
1.44	0.32	17.3	NA	NA	NA	80.82
1.54	1.96	99.4	2	101.4	2	83.08
1.69	0.93	42.9	0.81	37.3	-12.09	84.04
3.55	3.02	67.4	3.04	67.8	0.7	83.43
2	2.18	84.8	NA	NA	NA	81.82
2.23	1.74	60.8	NA	NA	NA	83.16
1.94	2.04	82.1	NA	NA	NA	82
1.52	0.99	47.4	1.22	58.4	23.2	78.95
2.3	1.88	63.1	1.97	66.1	4.8	80.18
2.65	4.09	119.1	4	116.5	-2.2	81.33
2.34	3.01	100.3	NA	NA	NA	81.48
2.13	1.07	35.4	1.09	36	1.9	88.43
2.19	3.14	100.6	NA	NA	NA	83.18
2.45	3.136	98.8	NA	NA	NA	81.33
2.68	4.05	116.5	NA	NA	NA	81.96
1.37	1.34	71.3	NA	NA	NA	80.99
1.44	2.04	102.7	2.14	107.7	4.9	78.95
1.68	1.1	51.1	1.13	52.5	2.7	81.78
2.51	2.54	78.2	2.7	82.1	6.3	79.23
1.56	1.61	80.5	1.62	81	0.6	82.33
2.19	2.68	94.4	2.61	91.9	-2.6	79.44
1.48	1.29	67.2	1.54	80.2	19.4	79.23
2.45	1.99	63.2	2.3	73	15.6	78.29
1.66	2.02	94.9	NA	NA	NA	81.03
1.49	1.29	62.9	NA	NA	NA	79.51
2.27	2.76	93.9	2.93	99.7	6.2	79.55
1.47	1.81	89.2	1.83	90.1	1.1	79.14
2.03	1.31	50.4	1.58	60.8	20.6	77.49
3.76	3.12	65.7	3.27	68.8	4.8	84.17
2.19	2.53	90	2.54	90.4	0.4	81.08
2.8	3.17	87.6	NA	NA	NA	79.86
1.72	2.16	97.7	NA	NA	NA	80.85
2.01	2.09	80.1	2.07	79.3	-1	79.55
3.8	4.43	92.3	NA	NA	NA	83.85
1.62	2.03	97.1	NA	NA	NA	80.08
2.75	4.43	124.2	NA	NA	NA	79.65
2.2	2.79	89.1	NA	NA	NA	84.31
3.07	3.43	88.4	NA	NA	NA	82.7
2.6	2.76	83.9	2.82	85.7	2.2	83.43
1.62	1.76	83.8	1.83	87.1	4	78.92

1.87	2.12	79.7	2.3	86.5	8.5	86.34
3.41	3.96	89.7	NA	NA	NA	81.75
2.01	2.49	87.4	2.43	85.3	-2.4	84.66
1.56	1.56	78	1.57	78.5	0.6	80.26
2.23	3.24	102	NA	NA	NA	85.82
2.28	2.4	81.9	NA	NA	NA	80.72
2.39	2.95	86.8	NA	NA	NA	86.02
1.94	1.74	70.2	NA	NA	NA	84.06
1.68	1.21	56	1.42	65.7	17.4	80.84
1.8	1.51	65.4	1.68	72.7	11.3	82.36
2.05	1.59	60.5	1.65	62.7	3.8	78.25
2.68	1.86	54.9	2.08	61.4	11.8	84.27
3.21	2.93	70.6	2.91	70.1	0.7	82.49
1.91	1.53	62.4	1.61	65.7	5.2	77.48
3.04	3.02	78.6	NA	NA	NA	82.8
2.28	2.11	71.5	2.24	75.9	6.2	80.07
2.55	2.18	66.1	2.29	69.4	5	80.39
1.48	1.1	54.2	1.15	56.7	4.5	77.64
2.27	2.82	87.4	NA	NA	NA	83.47
1.83	2.17	91.2	2.16	90.8	0.5	79.44
1.51	1.64	84.8	1.64	84.8	0	80.63
1.78	2.01	88	NA	NA	NA	83.11
2.08	2.67	98.9	NA	NA	NA	79.76
2.48	3.09	96	3.34	103.7	8.1	81.75
1.95	3.01	120.3	NA	0NA	NA	82.19
1.79	2.18	94.8	NA	NA	NA	79.16
1.63	2.02	87.1	NA	NA	NA	89.69
2.65	2.08	60.6	NA	NA	NA	80.6
1.92	1.94	70.9	NA	NA	NA	89.16
1.65	1.93	91	NA	NA	NA	81.68
2.12	2.28	83.9	2.2	81	-3.5	80.7
3	3.46	91.1	NA	NA		83.12
1.97	1.97	77.8	2	79	1.5	81.44
1.6	0.91	44	0.87	42.1	-4.4	78.81
1.68	0.57	26.1	0.63	28.9	10.5	78.5
1.96	2.56	101.9	2.67	106.3	4.3	79.18
2.45	2.6	74.7	2.46	70.7	-5.4	84.71
3.9	3.58	72.8	3.54	72	-1.1	84.06
1.7	0.87	39.8	NA	NA	NA	79.53
1.71	1.47	66.4	1.48	66.8	0.7	79.34
2.07	1.73	65.3	1.85	69.8	6.9	79.57
2.02	1.9	73.4	NA	NA	NA	79.94
2.15	2.83	92.7	NA	NA	NA	87.68
3.01	3.84	100.9	NA	NA	NA	82.91
2.14	3	98.7	NA	NA	NA	88.06
2.4	2.89	93.8	NA	NA	NA	82.23
1.56	1.62	81	1.66	83	2.5	80.26
1.37	1.69	94.9	NA	NA	NA	77.76
1.85	2.22	93.3	NA	NA	NA	81.99
1.37	1.69	94.9	NA	NA	NA	77.76
1.62	1.68	80.8	1.359	76.4	-5.4	79.52
1.7	1.81	83	NA	NA	NA	81.6
1.69	0.92	42	1.15	52.5	25	78.29
2.01	2.67	93.4	NA	NA	NA	87.29
2.51	4.41	123.2	NA	NA	NA	83.59
2.32	2.78	93.6	NA	NA	NA	82.23
2.25	3.43	106.9	NA	NA	NA	85.07
2.2	2.55	90.4	NA	NA	NA	82.78
1.87	1.91	71.8	2.08	78.2	8.9	86.34

2.02	2.63	101.5	2.72	105	3.4	84.07
3.35	4.79	113.2	NA	NA	NA	83.96
3.85	4.23	86.9	NA	NA	NA	83.85
3.54	5	111.6	5.09	113.6	1.8	83.64
1.8	2.03	87.8	2.12	91.7	4.4	82.08
2.05	2.65	90.7	2.99	102.4	12.8	86.36
1.99	3.24	114.4	NA	NA	NA	88.04
1.98	2.36	83.8	NA	NA	NA	84.28
2.19	2.75	88.1	NA	NA	NA	86.75
2.27	2.04	63.1	2.2	68	7.8	96.38
2.08	1.11	41.6	1.22	45.7	9.9	79.76
1.3	0.73	43.2	0.77	45.6	5.5	78.39
2.37	1.26	41.14	1.4	45.7	11.1	78.81
1.91	1.23	50.2	1.27	51.8	3.3	79.55
1.09	0.75	50	0.96	64	28	76.11
2.37	2.44	80.3	2.52	82.9	3.3	81.86
2.28	3.29	101.4	NA	NA	NA	85.44
3.24	3.46	82.4	NA	NA	NA	80.49
2.53	2.73	83.2	2.7	82.3	-1.1	80.28
2.08	3.02	102	NA	NA	NA	85.23
2.12	3	109.6	NA	NA	NA	79.76
2.01	3.05	117.3	3.14	120.8	3	80.49
1.82	1.91	81.6	2.19	93.6	14.7	81.61
2.95	3.27	85.4	3.47	90.6	6.1	81.54
2.97	3.03	78.7	3.62	94	19.5	81.86
1.4	1.42	73.7	1.36	70.6	-4.2	79.03
2.27	2.2	75.3	NA	NA	NA	80.15
2.04	1.93	73.7	NA	NA	NA	83.89
2.13	1.41	51.5	1.67	60.9	18.4	81.45
1.88	1.37	56.8	1.54	63.9	12.4	81.8
1.62	0.89	42.8	1.06	51	19.1	83.09
1.51	1.14	59.1	1.15	59.6	0.9	82.7
1.8	0.44	19.1	0.49	21.2	11.4	81.79
2.11	1.73	63.8	2.06	76	19.1	81.64
1.62	1.32	63.4	1.35	64.9	2.3	81.59
2.73	2.74	77.4	NA	NA	NA	81.23
3.01	3.87	101.8	NA	NA	NA	83.85
2.59	2.65	79.1	NA	NA	NA	80.07
2.83	3.39	92.6	NA	NA	NA	81.44
1.94	1.54	61.8	NA	NA	NA	84.63
2.62	3.57	105.3	NA	NA	NA	81.65
2.2	2.98	105.7	NA	NA	NA	82.78
1.95	2.31	91.4	NA	NA	NA	80.7
1.58	1.61	79.7	1.58	78.2	-1.9	81.02
3.16	3.74	91.5	NA	NA	NA	82.49
1.96	2.1	82.6	NA	NA	NA	80.28
1.89	1.96	80.7	2.17	89.3	10.7	83.5
1.91	1.96	80.1	2.18	89.1	11.2	79.55
1.84	1.51	64	1.54	65.3	2	81.23
2.13	1.57	57.3	NA	NA	NA	79.39
1.52	1.07	51.2	1.27	60.8	18.7	79.7
2	1.04	40	1.17	45	12.5	79.76
1.45	0.56	30.1	0.62	33.3	10.7	81.94
2.1	2.77	102.6	2.82	104.4	1.8	82.58
1.8	1.79	77.6	NA	NA	NA	81.51
1.84	1.73	73	NA	NA	NA	80.29
2.66	3.6	104.3	NA	NA	NA	81.86
3.84	4.16	85.6	NA	NA	NA	84.06

FEV1/FVC N	FEV1/FVC Pre	FEV1/FVC Pre %Prev	FEV1/FVC After
67.85	79.1	101.3	77.33
66.74	84.22	108.4	86.54
69.4	34.57	43.3	36.03
75.65	60.76	69	73.27
70.09	64.97	79.6	65.15
68.3	57.25	72.8	55.65
72.96	63.66	75.8	62.43
73.51	59.32	70.1	58.77
68.01	78.95	99.7	81.58
69.93	83.95	103.1	89.48
67.48	52.51	67.6	NA
70.57	62.23	75.7	67
73.41	86.68	101.4	90.14
70.28	77.03	94.2	79.2
73.2	71.31	83.7	71.04
70.31	73.62	91	81.21
67.83	80.46	101.9	NA
73.38	59.68	69.9	59.28
68.58	49.09	62.2	53.69
73.32	76.18	90.3	81.4
68.85	59.21	74.7	62.09
75.8	71.81	81.4	62.84
74.79	91.79	105.4	93.48
72.96	79.12	94.2	80.67
72.14	68.74	82.8	65.88
68.12	44	56.1	50.09
71.5	54.33	66	53.84
69.58	78.7	97.2	NA
69.03	51.29	64.6	52.25
69.82	60.08	73.9	65.54
68.18	51.2	64.5	53.29
71.86	82.14	99.3	85.43
72.22	84.35	100.3	85.27
76.09	92.68	104.6	94.88
74.15	90.85	105.2	94.96
74.81	67.27	77.2	69.56
71.1	78.54	94.9	NA
66.67	77.5	99.9	78.51
67.84	53.16	67.3	NA
68.21	57.01	72.6	58.54
69.26	74.29	92.1	71.3
70.59	55.45	68.3	61.31
71.69	63.82	76.5	NA
69.94	73.91	90.8	NA
73.37	82.47	96.9	86.92
70.25	57.11	69.8	66.98
72.55	61.88	73.3	61.24
72.72	72.62	85.8	72.71
67.98	60.02	75.8	61.4
69.22	75.05	94.2	77.95
70.23	49.62	60.7	47.83
74.18	76.47	88.5	82.59
68.31	80.95	101.8	82.47
68.3	52.55	66.1	59.17
69.86	64.96	80.8	67.21
69.13	52.9	66.5	56.7
69.95	84.43	103.7	81.94
71.37	68.05	81.9	66.65

70.23	84.22	103	87.19
75.5	81.21	92.4	90.35
71.2	71.86	86.7	71.24
74.96	75.13	86.1	77.15
68.7	81	101.3	80.4
72.96	68.24	81.3	84.7
71.86	77.21	93.4	79.38
69.58	80.08	100	85.89
69.13	47.9	60.2	51.44
73.69	86.46	102	90.46
71.58	49.9	59.9	59.14
73.41	69.02	81.7	71.93
70.11	45.2	55.4	43.13
71.04	73.06	89.4	76.03
68.85	68.13	86	NA
68.94	56.26	70.9	62.72
69.27	90.77	112.6	94.63
72.05	83.52	99.6	80.39
67.06	81.21	104	81.38
69.31	81.76	105.5	80.78
70.09	40.38	49.5	42.1
69.22	67.98	85.3	NA
70.49	84.23	103.8	NA
71.77	81.42	98.6	85.34
73.87	84.12	97.8	87.66
69.13	74.39	93.5	77.53
72.87	77.19	91	81.18
72.71	81.14	95.9	84.31
72.9	90.96	107.2	91.48
70.89	87.76	106.3	89.93
68.98	66.57	82.9	74.2
70.1	70.36	86.2	71.78
70.29	76.25	93.2	78.53
74	60.09	69.8	63.12
69.22	82.69	103.8	81.41
71.08	86.49	104.5	87.52
72.07	71.15	84.8	69.81
73.67	79.19	92.3	82.27
75.31	84.87	96.8	87.92
71.94	85.22	101.8	90
68.32	70.51	88.7	77.08
70.04	86.97	107.9	89.9
68.01	80.65	101.9	82.98
73.6	90.46	106.8	96.45
69.83	66.59	81.9	67.65
67.17	53.23	68.14	53.51
68.66	69.55	87	75.21
68	63.08	79.7	63.19
73.04	57.62	67.8	66.7
68.21	58.84	75	48.96
74.69	67.3	77.4	NA
73.87	65.14	75.7	69.14
72.41	53.85	64.6	52.89
70.06	64.74	79.4	NA
71.22	51.28	62.6	54.27
68.28	43.76	55.1	49.45
69.86	63.09	78.5	65.5
69.09	83.43	103.7	87.4
65.38	66.6	87.5	67.69

70.96	77.86	94.3	82.22
68.85	81.92	103.4	63.39
73.14	77.08	91.6	82.04
68.47	76.92	96.5	77.88
72.4	86.06	102.1	87.82
75.14	57.68	65.9	64.96
72.68	78.3	93.6	78.49
69.8	79.06	97.3	NA
73.41	59.66	70.6	59.41
68.99	74.45	92.7	69.8
71.13	85.65	104.6	86.84
73.55	90.5	105.7	92.11
68.51	79.55	99.7	NA
69.49	86.23	108	92.03
71.93	87.16	104.1	87.42
72.59	82.7	99	NA
69.78	77.42	95.3	79.4
69.13	89.75	112.8	89.46
68.03	65.47	83.6	70.16
69.42	35.11	43.4	NA
71.37	83.51	100.5	84.39
72.19	97.06	115.5	95.74
72.5	88.73	106.4	88.94
70.28	84.61	103.4	NA
71.43	76.56	92.1	NA
70.44	84.17	102.7	NA
67.82	60.98	77.2	68.96
69.67	60.51	75.5	56.6
70.68	79.45	97.7	79.26
69.99	87.58	107.5	NA
75.97	49.91	56.4	48.46
71.45	86.52	104	NA
70.68	79.83	98.2	NA
71.22	90.89	110.9	NA
69.57	83.65	103.3	NA
67.81	85.48	108.3	85.4
70.25	55.35	67.7	55.39
68.85	71.42	90.1	76.19
70.72	74.93	91	80.8
69.03	80.03	100.7	79074
68.85	55.06	69.5	59.01
67.25	69.46	88.7	74.35
69.6	82.52	101.8	NA
68.3	76.23	95.9	NA
69.13	77.51	97.4	78.54
67.98	77.06	97.4	79.9
66.57	58.75	75.8	67.1
73.14	78.25	93	80.47
69.65	88.75	109.5	91.57
69.4	79.76	99.9	NA
69.45	85.86	106.2	NA
69.13	74.54	93.7	69.55
72.87	88.88	106	NA
68.79	84.2	105	NA
69.22	87.59	110	NA
72.42	86.87	103	NA
71.86	71.36	86.3	NA
72.5	81.47	97.7	83.48
68.58	80.4	101.9	78.39

74.16	85.3	98.8	89.06
71.04	85.47	104.5	NA
72.72	83.21	98.3	85.03
68.95	79.51	99.1	77.29
73.72	91.96	107.2	NA
69.34	91.74	113.7	NA
73.89	85.92	99.9	NA
72.21	67.86	80.7	NA
69.44	68.31	84.5	68.8
70.75	73.18	88.9	76.82
67.22	69.09	88.3	68.43
73.23	59.42	70.5	59.86
71.68	75.36	91.4	75.78
66.56	70.74	91.3	71.03
71.95	90.97	109.9	NA
69.58	81.56	101.9	81.1
69.86	75.01	93.3	77.07
66.7	71.01	91.5	65.52
7107	88.52	106.1	NA
69.03	69.21	87.1	67.93
69.27	89.79	111.4	90.93
71.39	81.93	98.6	NA
69.31	77.76	97.5	NA
71.04	73.54	90	77.88
70.6	86.5	105.2	NA
68	76.03	96	NA
77.04	85.91	95.8	NA
70.04	86.43	107.2	NA
79.59	79.14	88.8	NA
70.17	92.7	113.5	NA
69.32	84.19	104.3	89.06
72.23	87.19	104.9	NA
69.95	77.23	94.8	83.1
68.49	50.08	63.5	47.65
68.21	41.46	52.8	43.34
68.02	67.4	85.1	70.85
72.77	83.97	99.14	85.41
73.05	89.97	107	90.04
68.31	85.34	107.3	NA
68.94	70.63	89	71.59
68.35	78.66	98.9	78.4
68.67	87.9	11	NA
75.32	85.52	97.5	NA
72.05	88.81	107.1	NA
75.64	86.67	98.4	NA
70.64	70.3	85.5	NA
69.85	71.01	88.5	69.86
67.57	71.42	91.8	NA
70.43	87.94	107.3	NA
67.57	71.42	91.8	NA
68.31	73.57	92.5	79.69
70.09	84.13	103.1	NA
68.03	36.88	47.1	39.97
74.98	82.79	94.9	NA
71.81	90.26	108	NA
70.63	62.63	100.5	NA
73.07	92.35	108.69	NA
71.11	80.05	96.7	NA
74.16	81.03	93.8	80.92

72.22	70.48	83.8	72.28
72.96	82.82	98.6	NA
72.387	83.76	99.9	NA
72.68	83.98	100.4	86.6
70.5	82.53	100.6	86.13
74.18	82.65	95.7	90.97
75.63	85.21	96.8	NA
72.4	72.55	86.1	NA
74.52	83.8	96.6	NA
74.2	78.41	90.8	83.8
68.51	60.26	75.6	67.36
68.12	42.21	53.8	42.95
68.49	46.12	58.5	47.04
68.33	73.84	92.9	75.49
65.38	58.12	76.4	57.14
70.31	63.76	77.9*	63.68
73.4	90.88	106.4	NA
69.95	80.56	100.1	NA
69.76	78.41	97.7	79.7
73.22	90.61	106.3	NA
68.51	79.83	100.1	NA
69.95	77.46	96.2	78.25
70.1	79.15	97	83.35
70.86	77.17	94.6	80.25
71.13	73.86	90.2	80.62
67.89	74.62	94.4	75.57
68.85	83.13	103.7	NA
72.06	74.24	88.5	NA
69.97	68.93	84.6	71.14
70.27	67.34	82.3	73.95
71.37	89.99	108.3	92.44
71.04	75.95	91.8	75.39
70.26	35.38	43.3	33.8
70.13	66.77	81.8	69.63
70.08	55.17	67.6	55.71
70.59	84.77	104.4	NA
72.87	85.96	102.5	NA
69.58	79.33	99.1	NA
70.77	83.04	102	NA
72.7	85.26	100.7	NA
70.95	75.68	92.7	NA
71.11	81.98	99	NA
70.132	83.65	103.7	NA
69.59	76.41	94.3	78.8
71.68	90.5	109.7	NA
69.76	76.99	95.9	NA
71.72	69.2	82.9	71.75
68.33	65.74	82.6	75.02
69.78	79.24	97.6	77.93
68.19	73.06	92	NA
68.46	68.76	86.3	68.92
69.31	41.9	52.5	39.65
70.39	38.61	47.1	37.02
70.94	85.33	103.3	90.07
70.02	80.03	98.2	NA
68.97	89.06	110.9	NA
71.13	77.84	95.1	NA
73.05	82.15	97.9	NA

FEV1/FVC after %Prev	FEV1/FVC %Des
99	-2.2
111.4	2.8
45.1	4.2
83.2	20.6
79.8	0.3
70.8	-2.8
74.4	-1.9
69.5	0.9
103	3.3
109.9	6.6
NA	NA
81.5	7.7
105.5	4
96.8	2.8
83.4	0.4
100.4	10.3
NA	NA
69.4	0.7
68	9.4
96.5	6.9
78.4	4.9
71.2	-12.5
107.4	1.8
96.1	20
79.4	-4.2
63.9	13.8
65.4	0.9
NA	NA
65.8	1.9
80.6	9.1
67.1	4.1
103.3	4
101.4	1.1
107.1	2.4
110	4.5
79.9	3.4
NA	NA
101.2	1.3
NA	NA
74.6	2.7
88.4	-4
75.5	10.6
NA	NA
NA	NA
101.8	5.4
81.9	17.3
72.8	-1
85.9	0.1
77.6	2.3
97.9	3.9
58.5	-3.6
95.6	8
103.7	1.9
74.4	12.6
83.6	3.5
71.3	7.2
100.6	-2.9
80.2	-2.1

106.6	3.5
102.8	11.3
85.9	0.9
88.4	2.7
100.5	0.7
100.9	24.1
96	2.8
107.3	7.3
64.7	7.4
106.7	4.6
71	18.5
85.1	4.2
52.8	-4.6
93	4.1
NA	NA
79.1	11.5
117.4	4.3
95.8	-3.7
104.2	0.2
101.3	-1.2
51.6	4.3
NA	NA
NA	NA
103.3	4.8
101.9	4.2
97.5	4.2
95.7	5.2
99.6	3.9
107.8	0.6
109	2.5
92.4	11.5
88	2
96	3
73.3	5
102.2	-1.5
105.8	1.2
83.2	-1.9
95.9	3.9
100.3	3.6
107.5	5.6
91.9	3.6
111.5	3.4
104.8	2.9
113.9	6.6
83.2	1.6
68.4	0.5
94.1	8.14
79.8	0.2
78.4	15.8
62.4	-16.8
NA	NA
80.4	6.1
63.5	-14.8
NA	NA
66.2	5.8
62.2	13
81.5	3.8
108.7	4.8
88.9	1.6

99.5	5.6
104	0.6
97.5	6.4
97.7	1.2
104.2	2
74.3	12.6
93.8	0.2
NA	NA
70.3	0.4
86.9	-6.2
106.1	1.4
107.6	1.8
NA	NA
115.1	6.6
104.4	0.3
NA	NA
97.7	2.6
112.5	0.3
89.6	21.1
NA	NA
101.6	1.1
113.9	-1.4
106.6	0.2
NA	NA
NA	NA
NA	NA
87.3	13.1
70.6	-6.5
97.5	0.2
NA	NA
54.8	-2.9
NA	NA
NA	NA
NA	NA
NA	NA
108.2	0.1
67.7	0.1
96.2	6.7
98.1	7.8
100.4	0.04
7405	7.2
95	7
NA	NA
NA	NA
98.7	1.3
101	3.7
86.6	14.2
95.6	2.8
112.9	3.2
NA	NA
NA	NA
87.4	-6.7
NA	NA
NA	NA
NA	NA
NA	NA
NA	NA
100.1	2.5
99.3	-2.5

103.2	4.4
NA	NA
100.4	2.2
96.3	-2.8
NA	NA
NA	NA
NA	NA
NA	NA
85.1	0.7
93.3	5
87.5	-1
71	0.7
91.9	0.6
91.7	0.4
NA	NA
101.3	0.6
95.9	2.7
84.4	-7.7
NA	NA
85.5	-1.8
112.8	1.3
NA	NA
NA	NA
95.3	5.9
NA	NA
NA	NA
NA	NA
NA	NA
NA	NA
NA	NA
110.4	5.8
NA	NA
102	7.6
60.5	-4.9
55.2	4.5
89.5	5.1
100.8	1.7
107.1	0.1
NA	NA
90.2	1.4
98.5	0.3
NA	NA
NA	NA
NA	NA
NA	NA
NA	NA
87	-1.6
NA	NA
NA	NA
NA	NA
100.2	8.3
NA	NA
51.1	8.4
NA	NA
NA	NA
NA	NA
NA	NA
NA	NA
93.7	0.1

86	2.6
NA	NA
NA	NA
103.5	3.1
104.9	4.4
105.3	10.1
NA	NA
NA	NA
NA	NA
97	6.9
84.5	11.8
54.8	1.8
59.7	2
94.9	2.1
75.14	-1.7
77.8	0.1
NA	NA
NA	NA
99.3	1.6
NA	NA
NA	NA
97.2	1
102.1	5.3
98.4	4
98.5	9.2
95.6	1.3
NA	NA
NA	NA
87.3	3.2
90.4	9.8
111.3	2.7
91.2	0.7
41.3	-4.5
85.3	4.3
68.3	1
NA	NA
NA	NA
NA	NA
NA	NA
NA	NA
NA	NA
NA	NA
NA	NA
97.3	3.1
NA	NA
NA	NA
85.9	3.7
94.3	14.1
95.9	-1.7
NA	NA
86.5	0.2
49.7	-5.4
45.2	-4.1
109.1	5.6
NA	NA
NA	NA
NA	NA
NA	NA
